# Development and validation of survival prognostic models for head and neck cancer patients using machine learning and dosiomics and CT radiomics features: a multicentric study

**DOI:** 10.1186/s13014-024-02409-6

**Published:** 2024-01-22

**Authors:** Zahra Mansouri, Yazdan Salimi, Mehdi Amini, Ghasem Hajianfar, Mehrdad Oveisi, Isaac Shiri, Habib Zaidi

**Affiliations:** 1 Division of Nuclear Medicine and Molecular Imaging, Geneva University Hospital, CH-1211 Geneva, Switzerland; 2 Department of Computer Science, University of British Columbia, Vancouver, BC, Canada; 3 Department of Nuclear Medicine and Molecular Imaging, University of Groningen, University Medical Center Groningen, Groningen, Netherlands; 4 Department of Nuclear Medicine, University of Southern Denmark, Odense, Denmark; 5 University Research and Innovation Center, Óbuda University, Budapest, Hungary

**Keywords:** Head and neck cancer, Machine learning, Survival analysis, Radiomics, Dosiomics

## Abstract

**Background:**

This study aimed to investigate the value of clinical, radiomic features extracted from gross tumor volumes (GTVs) delineated on CT images, dose distributions (Dosiomics), and fusion of CT and dose distributions to predict outcomes in head and neck cancer (HNC) patients.

**Methods:**

A cohort of 240 HNC patients from five different centers was obtained from The Cancer Imaging Archive. Seven strategies, including four non-fusion (Clinical, CT, Dose, DualCT-Dose), and three fusion algorithms (latent low-rank representation referred (LLRR),Wavelet, weighted least square (WLS)) were applied. The fusion algorithms were used to fuse the pre-treatment CT images and 3-dimensional dose maps. Overall, 215 radiomics and Dosiomics features were extracted from the GTVs, alongside with seven clinical features incorporated. Five feature selection (FS) methods in combination with six machine learning (ML) models were implemented. The performance of the models was quantified using the concordance index (CI) in one-center-leave-out 5-fold cross-validation for overall survival (OS) prediction considering the time-to-event.

**Results:**

The mean CI and Kaplan-Meier curves were used for further comparisons. The CoxBoost ML model using the Minimal Depth (MD) FS method and the glmnet model using the Variable hunting (VH) FS method showed the best performance with CI = 0.73 ± 0.15 for features extracted from LLRR fused images. In addition, both glmnet-Cindex and Coxph-Cindex classifiers achieved a CI of 0.72 ± 0.14 by employing the dose images (+ incorporated clinical features) only.

**Conclusion:**

Our results demonstrated that clinical features, Dosiomics and fusion of dose and CT images by specific ML-FS models could predict the overall survival of HNC patients with acceptable accuracy. Besides, the performance of ML methods among the three different strategies was almost comparable.

**Supplementary Information:**

The online version contains supplementary material available at 10.1186/s13014-024-02409-6.

## Introduction

Head and neck cancers (HNCs) account for around 5% of all malignancies, with 931,931 new cases and 467,125 (almost half of the incidences) deaths worldwide reported in 2020 [1]. The standard recommended treatment for HNC patients includes surgery and radiation therapy (RT) as adjuvant or concurrent with chemotherapy [2–4]. These patients’ leading causes of treatment failure and death are locoregional recurrences and distant metastasis [5, 6], which can affect overall survival. Although some prognostic factors, such as tumor location, age, stage, and human papillomavirus (HPV) status, are beneficial for prognostication, these patients still present with very poor prognoses [7, 8]. Even patients with similar prognostic factors may have different ultimate outcomes [9]. For those patients who undergo radiotherapy, dose delivery to the different parts of the tumor (necrotic and hypoxic) can be insufficient or non-uniform, which might influence tumor recurrence or residuals, leading to metastases and affecting patients’ outcomes. 3D dose maps obtained from treatment planning, contain information about the uniformity or inhomogeneity of dose distribution which can be predictive. Traditionally, this information is summarized into dose-volume histograms (DVHs), which proved to have limited predictive value [10]. As such, developing a reliable prognostic analysis and outcome prediction algorithm based on information from dose distributions in more effective way is an essential step in assisting personalized decision-making and treatment strategies.

The use of radiomics analysis as a noninvasive, fast, and cost-efficient approach to extract various image-based quantitative features has proven to be valuable for patient prognosis and outcome prediction modeling [4, 11, 12]. Radiomics has played an essential role in characterizing the internal structures of tissues, e.g., intratumor inhomogeneities that are becoming more widely recognized as a related factor in HNC prognosis [13–15]. Several studies have shown that using multi-modality fusion-based radiomic features from different medical imaging modalities, such as CT, MRI, and PET can significantly improve the predictive power of radiomics for other cancer types [16–19].

Implementing the radiomics concept on 3D dose distributions (called Dosiomics [20]) provided an opportunity to use the valuable predictive information hidden in the 3D dose distributions more effectively than DVHs.

While most studies investigated normal tissue complication prediction ability of dosiomics [20–29], few of them used radiomics and dosiomics for prognosis or outcome prediction [8, 30]. For instance, Lee et al. [30] used the Radiomic and Dosiomic features to predict weight loss in lung cancer patients after RT. They demonstrated that this analysis could improve the power of predicting weight loss as a prognostic factor and developing personalized treatment planning. Wu et al. [8] established a prediction model using radiomic and dosiomic features for locoregional recurrence in HNC patients who had received intensity-modulated radiation therapy (IMRT) and revealed that dosiomics improves the prognostic results.

However, to the best of our knowledge, previous studies barely integrated the dose distribution with one of the imaging modalities to predict prognosis or treatment outcome for HNCs using fusion-based features. In a recent study by Cai et al. [31], they trained a model for overall survival prediction and used different fusions of CT and dose distributions, reporting that fusion models outperformed single-modality models.

This study aimed to investigate the value of radiomic and dosiomic features extracted from GTVs on CT images as the primary modality used in RT treatment planning and dose distributions, in addition to image fusion of CT and absorbed doses for the prediction of survival in HNC patients who received IMRT. Moreover, utilizing multiple combinations of machine learning algorithms and feature selection methods, we explored the optimal combination suitable for our purposes. We considered the prediction of the overall survival of patients after treatment as the endpoint.

## Materials and methods

**Fig. 1:**
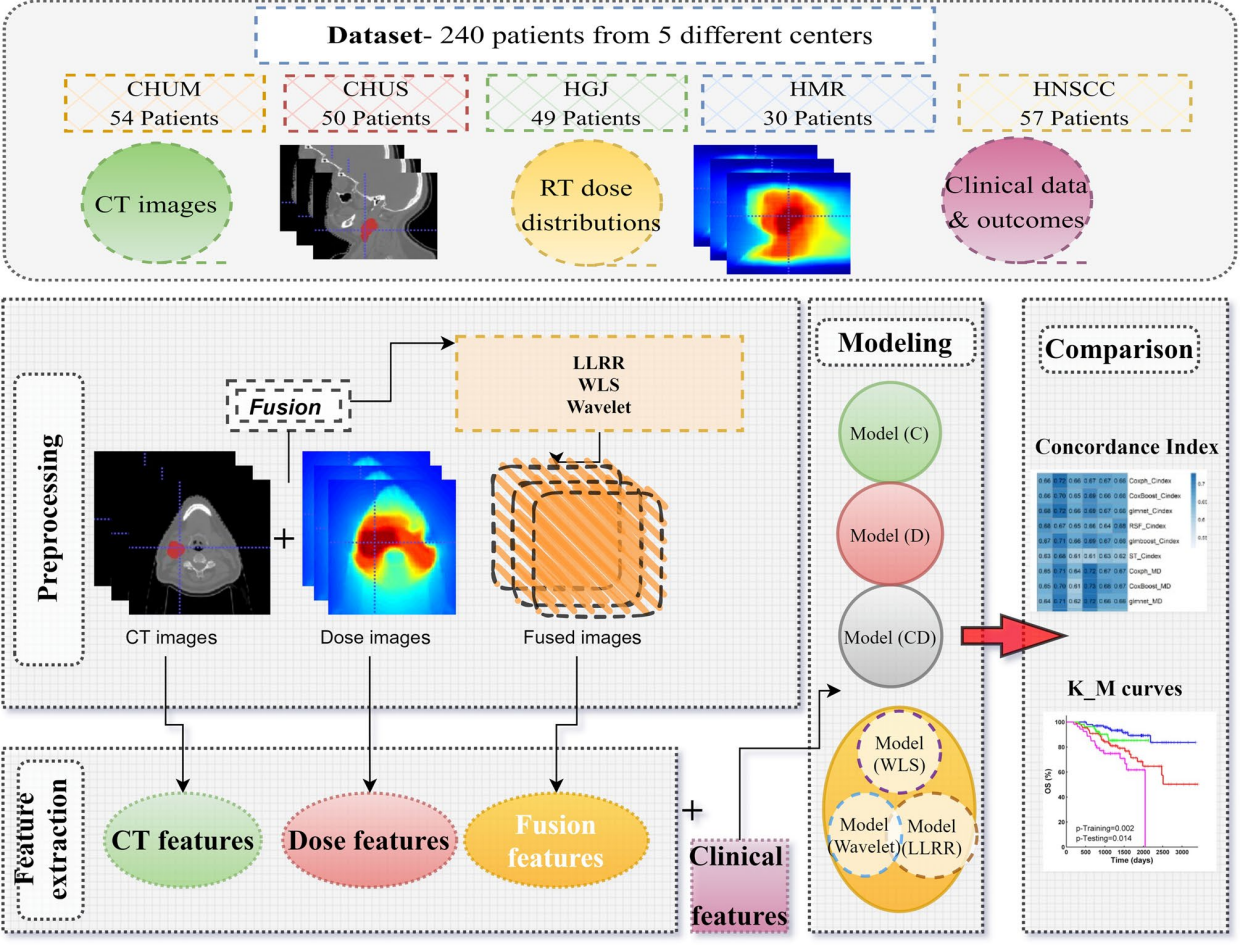
The flowchart adopted in this study protocol (radiomics, dosiomics, and three different fusion algorithms) combines dose and CT information to predict vital status and overall survival (time to event) for head and neck cancer patients

The overall workflow of the current study is shown in Fig. 1.

### Study population

A total of 240 patients with HNC obtained from the “Head-Neck-PET-CT” [32–34] and “HNSCC [35, 36]” databases archived in The Cancer Imaging Archive (TCIA) open access repository [32]. The “Head-Neck-PET-CT” database included data from four different centers, i.e., CHUM, CHUS, HGJ, and HMR, with 298 patients, whereas “HNSCC” included 627 patients. After excluding patients with incomplete data in terms of pre-treatment CT images, radiotherapy planning dose and outcome data, especially vital status, only 183 patients (54 CHUM, 50 CHUS, 49 HGJ, and 30 HMR) along with the other 57 patients were collected from “Head-Neck-PET-CT” and “HNSCC,” respectively, (in total 238 cases). The clinical characteristics of the analyzed patients are listed in Table 1. Overall survival of patients is defined as the time from diagnosis to the date of the last follow-up considered as the endpoint of this study. As evident in Table 1, the range of time from diagnosis to the last follow-up was 350–1806 days (average 1190 days), 245–2001 days (average 1189 days), 361–2119 days (average 1277 days), 194–2136 days (average 1195 days), 193–3542 days (average 2235 days) for CHUM, CHUS, HGJ, HMR and, HNSCC, respectively. For overall survival, our observation object was GTV which was contoured on CT images and stored in DICOM format retrieved from online datasets.

The metrics of age, sex, T-Stage, N-Stage, and TNM-group, primary tumor site, treatment type, and outcome were compared between the datasets with one way ANOVA test. P-values less than 0.05 were considered statistically significant.

### Preprocessing

All preprocessing procedures were performed in MatLab IBM (The Math Works Inc, *MATLAB*. Version 2020b) software. The dose distributions were registered on the axial CT images according to the location tag stored in the DICOM header. Then the GTV area was extracted and utilized for the next steps.

### Image fusion

To suppress any plausible bias in the results due to the selection of a specific image fusion model, three different publicly available algorithms were utilized to fuse CT images and dose maps. These included a technique based on 3D discrete wavelet transform, referred to as wavelet fusion (WF), one using visual saliency map (VSM) and weighted least square optimization, referred to as WLS, and finally, a fusion method based on latent low-rank representation referred as LLRR.

For the wavelet fusion [19], volumes (3D CT and dose maps) were first decomposed up to one level utilizing the wavelet basis function *symlet8* as a 3D discrete wavelet transform. Following the decomposition of volumes, which led to eight wavelet coefficient sub-bands for each volume, corresponding sub-bands were averaged to obtain a single set of fused sub-bands. Finally, fused wavelet coefficients underwent inverse 3D discrete wavelet transform to reconstruct the fused images.

For WLS fusion [37], first, a unique multi-scale decomposition (MSD) technique, including two filters, namely, a Gaussian filter and a rolling guidance filter (RGF), were applied to input images to decompose them into base and detail layers. With this specific MSD, information of the specific scales is maintained, and the voids near the edges reduce. For the fusion of the based layers, an enhanced VSM-based technique is used that suppresses the residual low-frequency information in based layers leading to better contrast and improved general visual appearance of the fused images. Detailed layers are merged by the state-of-the-art WLS optimization method, which captures more details and less noise. In the final step, fused, based and detailed layers are integrated to achieve the fused scan. The default parameters used by [37] were adopted in our study.

**Table 1:** Characteristics of patients included in this study protocols

Characteristics	CHUM	CHUS	HGJ	HMR	HNSCC	p-value
Total patients	54	50	49	30	57	-
Sex (M/F No.) (%)	38/16 70/30	32/18 64/36	40/9 82/18	24/6 80/20	48/9 84/16	0.089
Average Age (year)	62.1 ± 8.5	63.2 ± 11.6	62.2 ± 9.9	67.9 ± 9.9	56.7 ± 8.7	< 0.001
No. of patients >=60 years < 60	34 20	33 17	30 19	24 6	20 37	
T stage (No.) T1 T2 T3 T4 T4a T4b Tx	8 22 15 5 0 0 4	4 20 17 1 6 2 0	9 13 20 5 0 0 2	1 14 5 6 1 2 1	11 19 16 4 0 0 0	0.046
N stage N0 N1 N2 N2a N2b N2c N3 N3a N3b	3 7 38 0 0 0 6 0 0	18 4 25 0 0 0 3 0 0	10 10 0 6 15 7 1 0 0	5 4 0 0 10 9 1 0 1	10 9 2 2 24 8 2 0 0	< 0.001
TNM stage I II III IV V VI IVA IVB IIB IV1A	0 1 52 0 0 0 0 0 0 1	2 7 9 0 0 0 27 5 0 0	1 2 19 0 0 0 25 1 1 0	0 2 5 0 0 0 17 5 1 0	2 2 13 0 0 0 38 2 0 0	< 0.001
Primary site Unknown Oropharynx Nasopharynx Larynx Hypopharynx	4 47 2 0 1	0 36 3 10 1	2 34 5 6 2	0 14 4 7 5	2 47 2 4 2	< 0.001
Therapy Radiation only CHRT Surgery + RT Surgery + CHRT	3 51 0 0	15 35 0 0	3 46 0 0	5 25 0 0	9 26 5 17	< 0.001
RT modality	IMRT/TOMO	IMRT	VMAT	IMRT	IMRT	
Total prescribed dose (Gy, (median ± SD)	70 ± 1.6	69 ± 21	71 ± 2	68 ± 3.1	70 ± 2.3	
Outcome VS(alive/dead)	50/4	41/9	44/5	16/14	40/17	< 0.001
Time (days)* Average Min Max	1190 350 1806	1189 245 2001	1277 361 2119	1195 194 2136	2235 193 3542	

CHRT: Chemoradiation, VS: Vital Status, *diagnosis to last follow-up

For LLRR fusion [38], input scans were first fed into latent low-rank representation to decompose into two parts: the low-rank part, i.e., global structure, and the saliency part, i.e., detailed local structures. A weighted average strategy was used to fuse the corresponding low-rank parts to capture more edge information. Saliency parts were simply summed. The final step included the integration of the fused low-rank and saliency parts. In this fusion also, we used the default parameters presented by [38]. All image processing and image fusions were performed in Matlab®.

### Feature extraction

All images were interpolated to an isotropic voxel spacing of 1 × 1 × 1 mm^3^ prior to feature extraction, first to standardize the voxel size over images from different scanners/centers and second to preserve the rotational invariance characteristic of the texture features. In addition, the intensity levels inside ROIs were discretized to a 64-level grayscale to make the feature calculation tractable. A feature extraction package based on MATLAB®, known as the Standardized Environment for Radiomics Analysis (SERA)^1^ [39], was used for feature calculation. SERA agrees with guidelines from Image Biomarker Standardization Initiative (IBSI) [40]. This package was previously evaluated in multi-center standardization studies for improved feature reproducibility and robustness [40, 41]. Overall, 215 features per modality were extracted, i.e. 215 for CT, 215 for dose, and 215 for each fusion method. The feature set included 29 shape (namely morphological), 50 first-order (namely statistical, histogram and intensity histogram) and 136 three-dimensional texture features calculated using GLCM, GLRLM, GLSZM, GLDZM, NGTDM, and NGLDM matrices. Besides, 7 clinical features (age, sex, primary tumor site, T staging, N staging, TNM staging, treatment modalities) were also included alongside the other features to construct the prediction models. Noteworthy, one of the implemented strategies (Dual-CT-Dose) involved concatenating the radiomic (*n* = 215) and dosiomic (*n* = 215) features. This concatenated group, along with the inclusion of 7 clinical features, yielded a total of 437 features. Subsequently, feature selection was performed as described below. The details of the extracted features can be found in Supplementary Table 1.

### Feature selection

We utilized five distinct feature selection (FS) algorithms, namely C-Index, Minimal Depth (MD) [42, 43], Variable hunting (VH) [42, 43], Variable Importance (VH. VIMP) [42, 43], and Mutual Information (MI) [44] to identify appropriate features. The Concordance index for each feature was calculated using the C-Index FS method—a hybrid approach employing a filter and a wrapper based on univariate Cox proportional hazard regression. This calculation was performed after eliminating features in pairs with a Spearman’s rank correlation coefficient (rho) less than 0.9. Features with rho greater than 0.9 were retained for further analysis and subjected to a univariate Cox proportional hazard model. The top ten features demonstrating optimal performance (highest mean C-index) were selected through 100 repetitions using bootstrap resampling.

In MD, a method based on random survival forest, the features were sorted by depth, and those closer to the root node, indicating higher predictive power, were chosen. The top 10 features with minimal depth were selected. Notably, the number of features equal to 10 was an arbitrary choice based on the most predictive features (depending on the feature selection approach).

For other FS methods, such as VH and VH.VIMP, both model-based FS techniques utilizing the Random Survival Forest (RSF) model, the data were randomly divided into train and test sets. RSF was applied to the train set, and random features were selected based on the minimal depth threshold. The initial model was constructed using these selected features, with continiously adding the features until the importance of the joint variable is stabilized. This process was iterated 50 times, and the features with the highest frequency of occurrence were selected. It is noteworthy that the process for VH and VH.VIMP is identical, except for VH.VIMP, where variable importance is utilized for feature ordering, whereas VH relies on the minimal depth threshold—a method slower than VH.VIMP [42, 43, 45]. MI represents a completely parallelized implementation designed for computing the Mutual Information Matrix. The calculation of MI involved a linear approximation based on Pearson’s or Spearman’s correlation between two columns. To assess the correlation between survival data, Somers’ Dxy index was employed [44].

In supplementary Table 2, we provide detailed names of the features selected throughout the five-fold cross-validation.

### Time-to-event survival models machine learning and hyperparameter optimization

In this study, we assessed the performance of six machine learning algorithms that can manage the continuous time-to-event survival data. The models are listed below:

1. Cox model fitted the by likelihood-based boosting (CoxBoost) [46];
2. Random survival forest (RSF) [47];
3. Cox proportional hazard (Cox PH) [48];
4. Gradient boosting with a component-wise linear model (glmboost) [49];
5. Lasso and Elastic-Net regularized generalized linear model (glmnet) [50];
6. Survival tree (ST) [51].

**Fig. 2:**
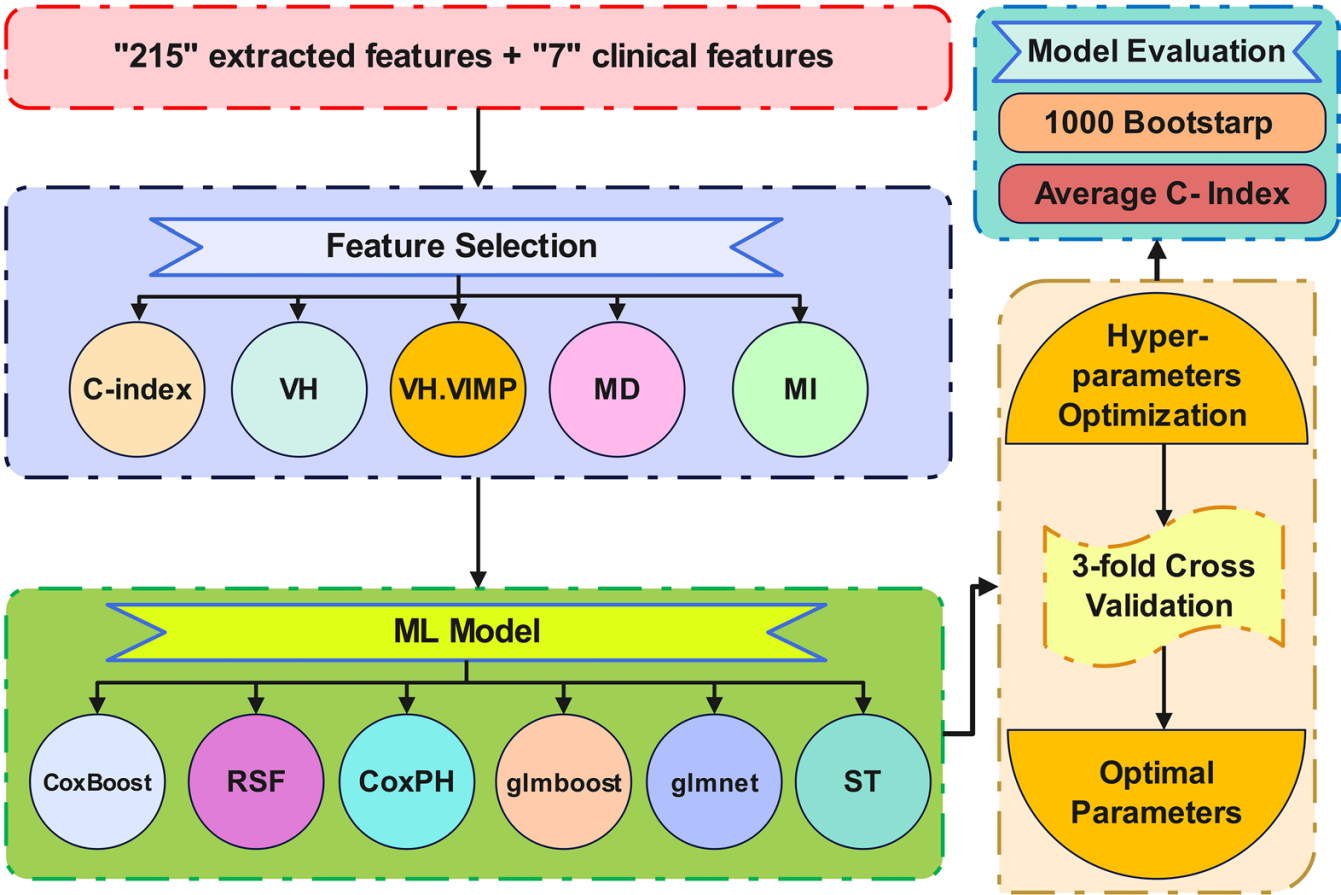
Flowchart describing feature selection, model training, model tuning, and model evaluation

Feature selection, all ML model training, model evaluation, and hyperparameters tuning were implemented in the MLR package in R programming language, version 3.6.2. The hyperparameters were tuned for all ML methods (except Coxph) using grid search. The details of hyperparameter settings and the R packages used in this study are mentioned in supplementary Table 3. The hyperparameter setting was guided by the C-index as a performance metric, calculated by 3-fold cross-validation in the training dataset. Figure 2 describes the feature selection, model training, hyperparameters tuning, and model evaluation.

### Model evaluation

This study used a one-leave-center-out strategy for model building and testing with a hold-out external validation set to build a generalizable model across the variability of centers, scanners, acquisition, reconstruction and treatment parameters. The feature normalization function was transformed from the training to the test set, meaning that the same normalization was implemented for features in both train and test dataset. After selecting features and optimized hyperparameters for ML models, ML models were tested on the hold-out external test dataset utilizing bootstrapping resampling with 1000 repetitions. This step was repeated five times, where in each time, one of the five centers selected as the test set and the remaining four centers as the training set. The results of the one-leave-center-out scheme were reported for all centers on average and for each center separately. C-indices were reported along with the standard deviations (± SD) among five scores from leave-one-center-out cross validation. With this methodology, we used all data sets as training and external hold-out test sets, which revealed model generalizability.

Kaplan-Meier curves were created for the best models, the cut-off criteria for risk stratification were the median of the risk scores calculated by the models, meaning the individuals witha risk score above or equal to and below the median were categorized into high-risk and low-risk groups, respectively. The log-rank test was used to calculate the p-values.

## Results

### Study population Anova test results

The ANOVA p-values were less than 0.05 when comparing all age, sex, T-Stage, N-Stage, and TNM-group, primary tumor site, treatment type, and outcome metrics among the five datasets, confirming that there is statistically significant difference amongst the population included in the cohort in terms of the considered metrics. The p-values are indicated in Table 1.

### Overall comparison between the different strategies

**Fig. 3:**
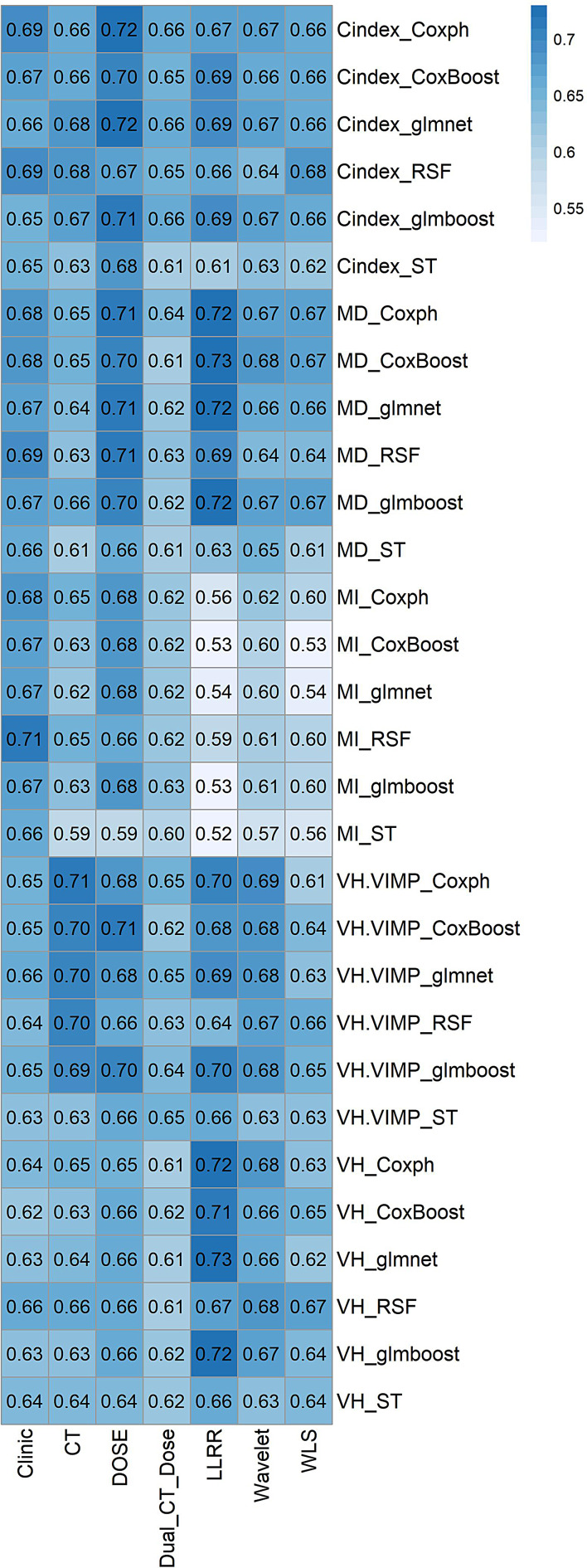
Heat map of C-index for each strategy and ML in combination with feature selection methods

Figure 3 depicts the heat map of the mean C-index for each strategy of radiomics, dosiomics, and fusion-based methods amongst all 30 combinations of feature selection and ML models. More information is detailed in supplementary Fig. 1, where the heatmaps are denominated with the name of the hold-out center (selected as test set).

**Fig. 4:**
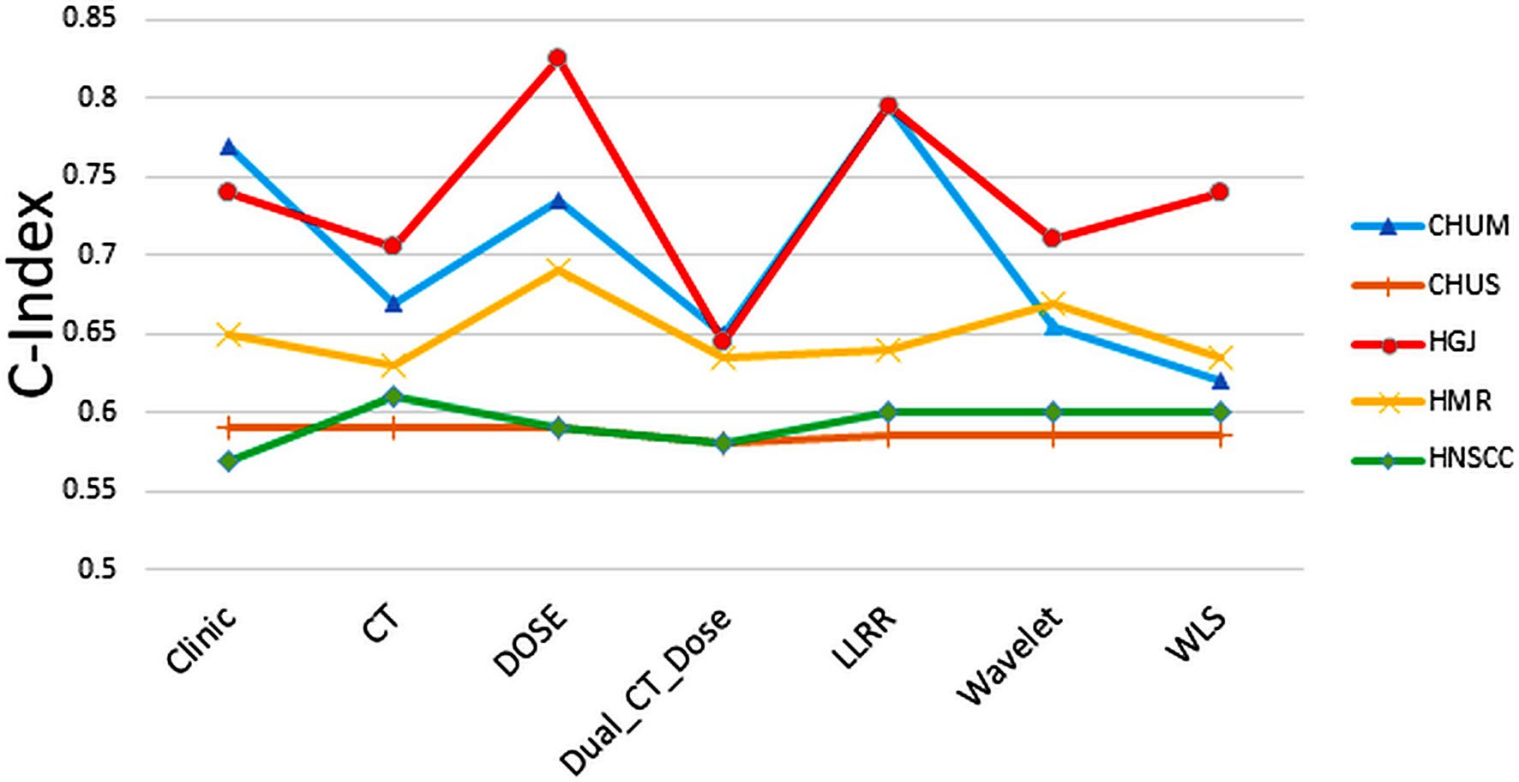
The median value of C-indices among all strategies and all models for CHUM (blue), CHUS (orange), HGJ (red), HMR (yellow), and HNSCC (Green)

**Fig. 5:**
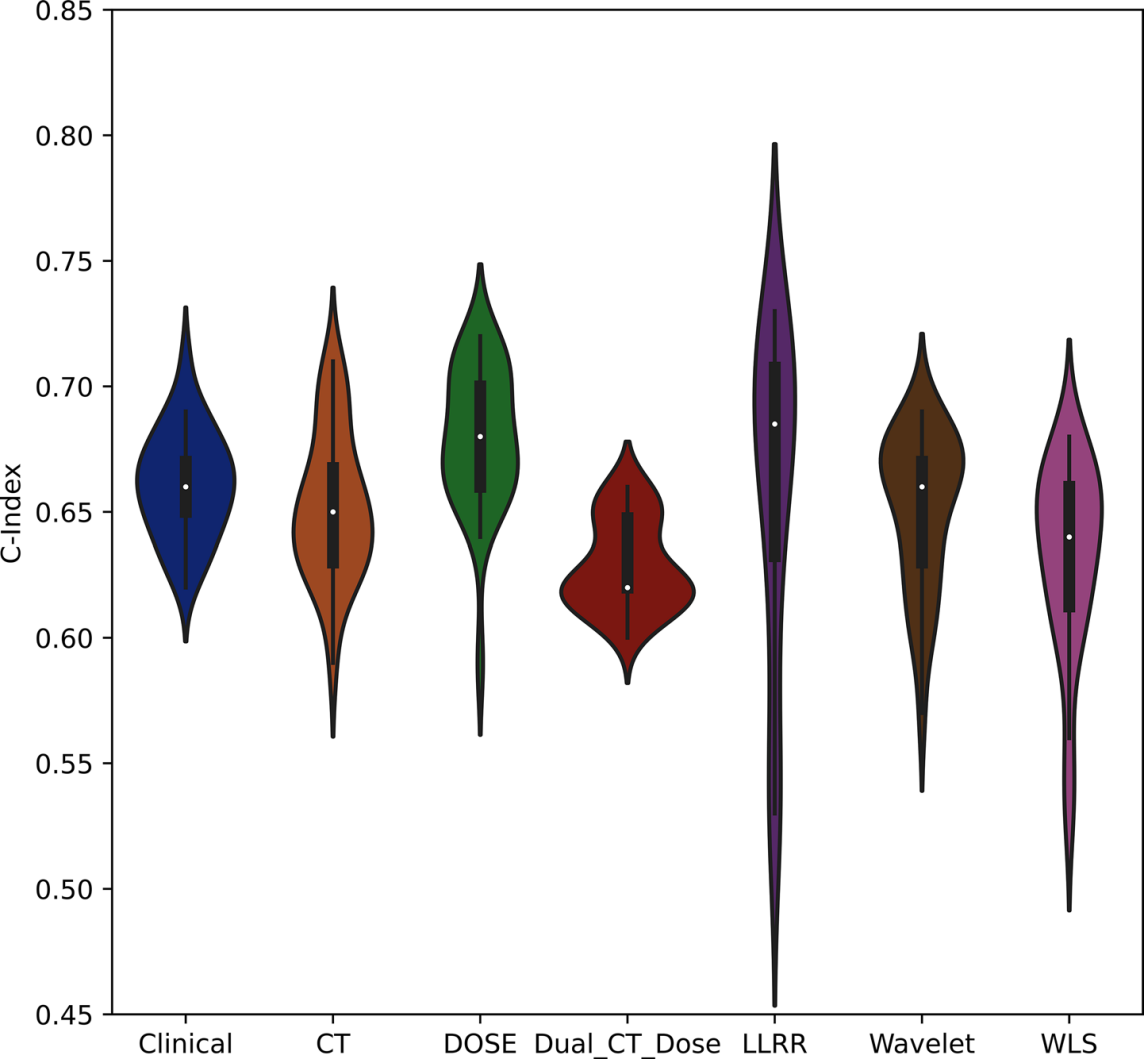
The violon plots of mean C-index for radiomic and dosiomic and three different fusion-based strategies for survival prediction

Figure 4 shows the median value of the CIs among all models and strategies. Moreover, The mean and SD of C-indices are also listed in Supplementary Table 4. The violon plots of Fig. 5 depict the distribution of mean C-indices for each strategy separately.

To evaluate the significance of differences among the different strategies, we performed Friedman test followed by Nemenyi post-hoc test as the P-value for the Friedman test was < 0.05. Supplementary Table 5 summarizes the results of Nemenyi post-hoc test, which showed significant difference among the different strategies.

### Comparicon of different machine learning models

For survival prediction, two model and feature selection methods, i.e., CoxBoost-MD (C-index = 0.73 ± 0.15) and glmnet-VH (C-index = 0.73 ± 0.15) for LLRR, achieved the highest performance relative to other features. In a comparison of the top 10% C-indices among all non-fusion strategies (Clinical, CT, Dose, and Dual-CT-Dose), the dose strategy (dosiomics) showed the highest values of C-indices (0.7–0.72). The comparison among fusion-based strategies (LLRR, Wavelet, WLS) revealed the highest values for LLRR (0.7–0.73). The minimum CI values among all models and all strategies were from ST_MI (0.52–0.6), except for WLS, in which the minimum CI was from CoxBoost-MI (0.53) but still among the minimum values, CI of ST-MI was significantly low (0.56).

**Fig. 6:**
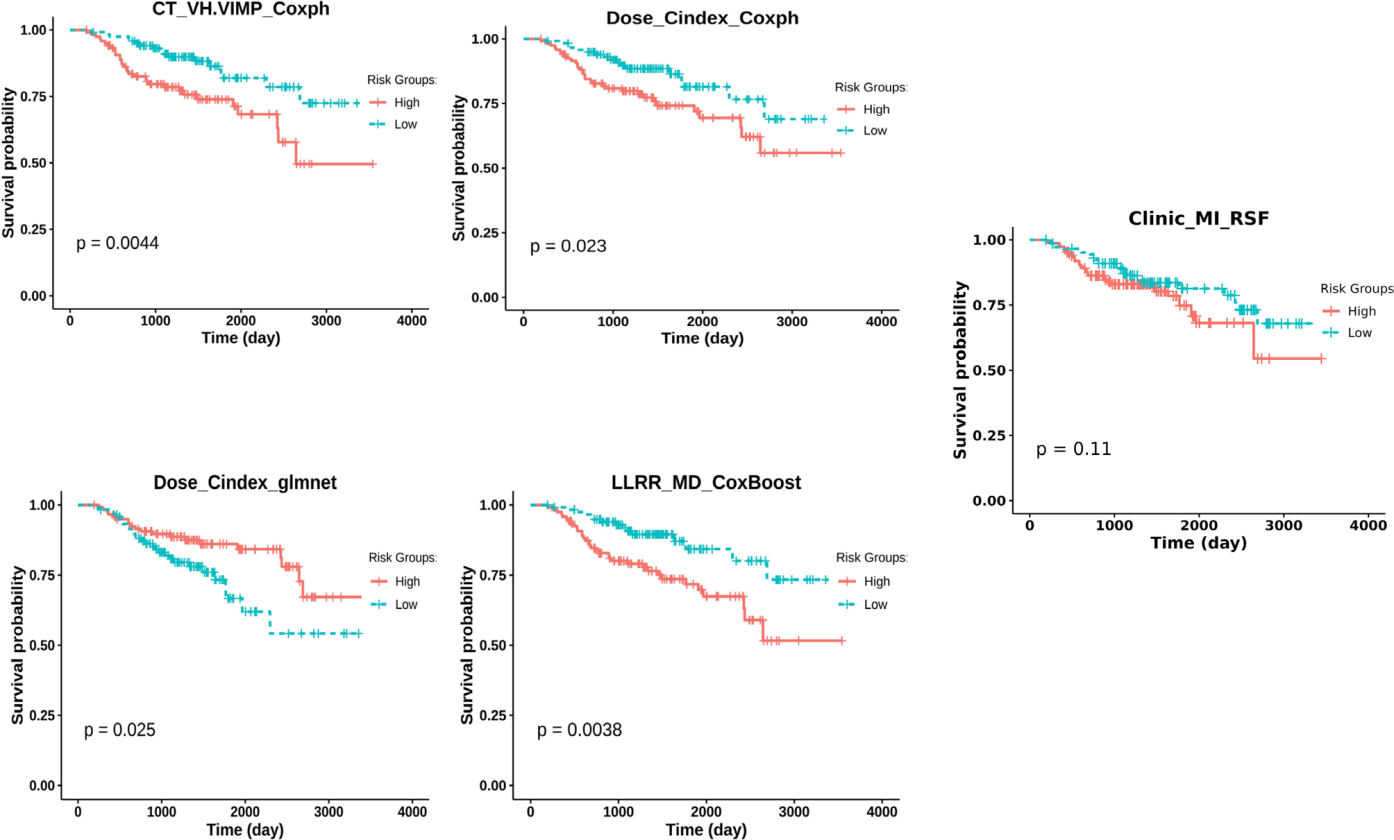
Kaplan-Meier curves of the high- and low-risk groups for the five best models by clinical, Dose and LLRR fusion strategy. The cut-off criteria for risk stratification were median. A log-rank test was used to calculate the p-values

Table 2 shows the highest mean C-index for the best machine learning and FS combinations and their corresponding strategy. All models have been assessed in one leave center out cross-validation. The corresponding CI values for each center are reported in supplementary Fig. 2 in separate heat maps, and the top 10% C-indices for any strategy are reported in Supplementary Table 6. The models with the highest CI values were selected as more efficient models, and the K-M curves were applied to them for further comparison. Figure 6 illustrates the K-M curves for the best models, showing statistically significant power in dividing groups into low and high risks. The other K-M curves for the best models for all strategies are shown in Supplementary Fig. 3.

**Table 2:** Best combinations of feature selection and machine learning methods (highest mean CIs) for each strategy. Standard deviations of CIs are also provided here and in the supplementary Table 3. Slash “/”separated SDs related to their corresponding feature selections

ML model	Feature selection method	Highest CI ± SD	Strategy
Coxph	CI/ MD/ VH	0.72 ± 0.14 / 0.15 / 0.15	DOSE/LLRR/LLRR
CoxBoost	MD	0.73 ± 0.15	LLRR
glmnet	VH	0.73 ± 0.15	LLRR
RSF	MD	0.71 ± 0.11	DOSE
glmboost	MD/ VH	0.72 ± 0.15 / 0.14	LLRR

### Comparison of different feature selection methods

A comparison between CI values in terms of evaluation of the feature selection methods performance showed that the higher values of CIs obtained from MD (0.73 ± 0.15 and 0.71 ± 0.11 for LLRR and Dose, respectively) and VH methods (0.73 ± 0.15 for LLRR). VH.VIMP method for dosiomics only (Dose) and radiomics only (CT) showed similar performance (0.71 ± 0.11). Among the fusion-based strategies, LLRR had the highest value (0.7 ± 0.12). In the C-index method, the most potent CI among non-fusion strategies belonged to dose image selected features, i.e., dosiomics (0.72 ± 0.14). In contrast, LLRR (0.69 ± 0.13) had the highest C-index value amongst the fusion-based methods.

## Discussion

In this study, we considered the image-based fusion of CT images and 3D dose distributions and integrate the concepts of radiomics and dosiomics by comprehensively comparing different machine learning and feature selection combinations to predict the overall survival of HNC patients after radiotherapy treatment. For this purpose, we used three different fusion algorithms to fuse the CT and dose maps. Then, corresponding extracted features were driven into the 30 different combinations of feature selections and machine learning algorithms to explore the value of dosiomics and fusion-based features. We also compared the results with single modality radiomics models. Our results demonstrated that the 3D dose distribution included valuable information highly correlated with overall survival prediction in HNC patients. Moreover, fusion (especially with the LLRR algorithm) of the dose distribution with CT images can improve some prediction models’ performance and accuracy. However, the fusion approach provided a slightly more accurate performance than the dosiomics approach alone.

This study was conducted under the assumption that in image-level fusion, the incorporation of neighboring voxel values in the fused images creates a novel texture that offers increased prognostic value compared to the interpretation of voxel values in an individual image alone (e.g. HU in CT or Gy in dose map), which may lack a clear physical or biological meaning.

Five independent datasets were used in this study. We carried out a one-way ANOVA statistical test on our cohort. According to the p-values reported in Table 1, there is a significant heterogeneity in population characteristics, such as sex and TNM staging, indicating that our models presented a robust behavior against the heterogeneity of characteristics in the cohort, indicating the generizability of the models. Moreover, the effect of cycling in hold-out dataset on the performance of the models was investigated during the one-center-leave-out procedure. It is worth emphasizing that our models were consistently trained incorporating clinical features. Specifically, the features derived from CT, Dose, Dual-CT-Dose, and fusion-based strategies were not exclusively image- or dose-based. Instead, clinical features were consistently integrated. In addition, we conducted analyses with models utilizing only clinical features to establish a baseline assessment. The results indicated that clinical strategies achieved a performance comparable to Dose and fusion levels in terms of the C-index. However, as depicted in Fig. 4, the median values of Dose and LLRR in the folds exhibited superior performance compared to the clinical approach. Furthermore, the clinical strategy did not perform effectively in stratifying high and low-risk cases based on the survival curves illustrated in Fig. 6.

It should be noted that we compared our results with models established by Wu et al. [8]. However, they investigated local recurrence prognostic models, whereas our models predicted the overall survival (OS), and as such, the results are not directly comparable. Still, this was the most similar study involving Dosiomics results in HNC patients. By comparing our results to Wu et al. [8] models, our established models outperformed (CI = 0.66 vs. 0.54) for CT with the Coxph-Cindex model and (CI = 0.72 vs. 0.66) with Coxph-CIndex for Dose. However, we implemented much more comprehensive ML-FS (6 × 5) methods and models evaluated in the multi-center strategy. The VIMP model for CT has shown an even higher CI (0.71). We also achieved a CI of 0.72 for the Dose model with glmnet-Cindex. Our training method has benefited from the one-leave-center-out scheme. However, Wu et al. [8] used the data from two centers for training and the other two centers for testing their models.

Moreover, in this study, we fused CT images and Dose distributions. By combining the radiomics and dosiomics data into a single fused image, the performance of specific models could be improved (CI increasing to 0.73 in glmnet-VH and CoxBoost-MD for the LLRR fusion method). Compared with a fusion-based radiomics study by Lv et al. [18], our results have shown a higher prognostic performance for OS (CI = 0.67 vs. 0.64) in the wavelet fusion strategy. They only implemented the Coxph-Cindex model with different fusion strategies. In contrast, our results for the same fusion strategy demonstrated that other ML-FS models have even better performance (the highest CI of 0.69 was achieved for the Coxph-VH. VIMP model in the wavelet fusion strategy). Besides, they fused PET and CT images of HNC patients. While in this study, we fused CT images and 3D dose distributions to combine their information in a single image. The average CI for the “CT only” strategy in this study with the Coxph-Cindex model was 0.66, whereas the CI of 0.71 was the highest prognostic performance using the Coxph-VH. VIMP model. However, the established model by Lv et al. [18] study achieved a CI of 0.59 ± 0.5.

In comparison with Vallières et al. [33] results, who used a random forest model, our RFS models showed a lower CI for OS (0.7 for CT Via RSF-VH. VIMP and 0.71 for Dose Via RSF-MD vs. 0.75 for CT and 0.76 for PET). It should be noted that the larger number of extracted features in their study (1615 vs. 215 + 7 in our study) may have influenced their results. In a CT-based radiomics study by Sun et al. [52] in which the effect of ML methods on lung OS was investigated, the highest reported CI for some ML models were similar to some of ours, i.e., 0.674, 0.627, and 0.646 for CoxBoost-Cindex, RSF-PCC and Cox-MI, respectively. By including the dose information (dosiomics) and fusing the dose with CT images, the performance improved compared with the dose and CT-only approach (0.7 for Dose via CoxBoost-Cindex, 0.71, 0.69, for Dose, and LLRR, respectively, via RSF-MD, 0.68 via RSF Cindex and RSF-VH for WLS and Wavelet respectively and 0.68 via Coxph-MI for Dose strategy).

In a study by Lee et al. [30], the authors considered a radiomics and dosiomics strategy to predict the weight loss of lung cancer patients after radiotherapy. To summarize, a review of the dosiomics results demonstrated that our most robust dosiomics models (CI of 0.72, Coxph-Cindex, and glmnet-Cindex) are as predictive as theirs (AUC = 0.71). It should be noted that AUC and CI are not directly comparable, and the subjects used in these studies are different.

In general, our models showed a good performance, particularly when using features derived from 3D dose distributions (Dosiomics). According to our findings, the combination of radiomics and Dosiomics into a single fused image (particularly employing the LLRR approach) yielded comparable results to those achieved with Dosiomics alone. However, Dosiomics remains unaffected by differences in CT characteristics. Moreover, the approach is both methodologically and conceptually simpler, requiring less data for collection.On the other hand, an investigation involving larger cohorts may reveal an increased discrimination between Dosiomics and fusion-based models as the hypothesis is that such models would derive greater benefits from a large-scale dataset.

The combination of LLRR fusion and specific feature selection and machine achieved the highest average C-Index. However, this doesn’t mean that LLRR fusion always performs better with all feature selections/machines. We suggest that the model achieving the best performance be used. However, if there is a desire to test the model with limited inputs, such as clinical only, CT only or Dose only inputs, the best model according to supplementary Table 6 should be selected and used.

A radiotherapy 3D dose map contains by nature information correlated to outcome prediction. Although dose-volume histograms are the most common tools to display this information, this information is accumulated in existing DVHs, and DVHs are deficient in showing the spatial information [53–55]. Using the DVH-based metrics may even over/underestimate the prediction of therapeutic toxicity in head and neck cancer radiotherapy by up to 50% [56].

This study provided information to tailor a subset of feature selection and ML algorithms for overall survival prediction modeling in HNC patients. While this study involved five independent centers and multiple treatment modalities, a major limitation was the limited sample size and the usage of non-harmonized feature-sets. To mitigate the limited size, we utilized a one center leave-out strategy and averaged the results to enhance the reproducibility across different centers. However, further studies using larger databases and implementing more robust harmonization methods are still required. We showed that Dosiomics could more robustly explain the features within the 3D dose maps. Moreover, the results were extended to the fusion of treatment planning CT and radiotherapy dose distributions. Overall survival prediction based on radiomics and dosiomics and the fusion of these two images can be helpful in the decision-making process and personalized treatment.

## Conclusion

We proposed a comprehensive framework for the development and validation of time-to-event overall survival models (cross-combination of feature selection and ML) for clinical as baseline, single (CT, Dose), multi-modality (CT-dose) and fusion models (Wavelet, LLRR, WLS). We also investigated the best combination of feature selection and machine learning model reported for each strategy. Our results demonstrated the potential of clinical, radiomics applied on CT, dosiomics derived from radiotherapy 3D dose distributions, and three different fusion strategies in overall survival prediction of head and neck cancer patients. Our results support the superiority of dosiomics in identifying prognoses associated with overall survival. The fusion-based models showed a comparable result to dosimics tending to improve the results of overall survival prediction probably by training models with large-scale datasets.

### Electronic supplementary material

Below is the link to the electronic supplementary material.

Supplementary Material 1

## Data Availability

The data are available from The Cancer Imaging Archive (TCIA) open access repository.

## Footnotes

1 https://github.com/ashrafinia/SERA.

## Abbreviations

CT: Computed Tomography
PET: positron emission tomography
GTV: gross tumor volumes
Dosiomics: dose distributions
HNC: Head and neck cancer
FS: feature selection
ML: machine learning
CI: concordance index
OS: overall survival
MD: Minimal Depth
VH: Variable hunting
LLRR: latent low-rank representation referred
RT: radiation therapy
DVH: dose volume histogram
IMRT: intensity-modulated radiation therapy
TCIA: The Cancer Imaging Archive
WF: wavelet fusion
VSM: visual saliency map
MSD: multi-scale decomposition
RGF: rolling guidance filter
