# Supplementary material for: Development and validation of survival prognostic models for head and neck cancer patients using machine learning and dosiomics and CT radiomics features: a multicentric study
Source: Radiat Oncol. 2024 Jan 22;19:12. doi: 10.1186/s13014-024-02409-6 (PMC10804728; doi:10.1186/s13014-024-02409-6)
Supplement: Supplementary file 1 — Supplementary Material 1 [file 13014_2024_2409_MOESM1_ESM.pdf]

Supplementary

Supplementary Table 1. Detailed list of the extracted radiomic features.

	family	image_biomarker	Tag
**1**	Morphology	Volume (mesh-based)	morph_volume
**2**	Morphology	Volume (counting)	morph_vol_approx
**3**	Morphology	Surface area	morph_area_mesh
**4**	Morphology	Surface to volume ratio	morph_av
**5**	Morphology	Compactness 1	morph_comp_1
**6**	Morphology	Compactness 2	morph_comp_2
**7**	Morphology	Spherical disproportion	morph_sph_dispr
**8**	Morphology	Sphericity	morph_sphericity
**9**	Morphology	Asphericity	morph_asphericity
**10**	Morphology	Centre of mass shift	morph_com
**11**	Morphology	Maximum 3D diameter	morph_diam
**12**	Morphology	Major axis length	morph_pca_maj_axis
**13**	Morphology	Minor axis length	morph_pca_min_axis
**14**	Morphology	Least axis length	morph_pca_least_axis
**15**	Morphology	Elongation	morph_pca_elongation
**16**	Morphology	Flatness	morph_pca_flatness
**17**	Morphology	Volume density (AABB)	morph_vol_dens_aabb
**18**	Morphology	Area density (AABB)	morph_area_dens_aabb
**19**	Morphology	Volume density (OMBB)	morph_vol_dens_ombb
**20**	Morphology	Area density (OMBB)	morph_area_dens_ombb
**21**	Morphology	Volume density (AEE)	morph_vol_dens_aee
**22**	Morphology	Area density (AEE)	morph_area_dens_aee
**23**	Morphology	Volume density (MVEE)	morph_vol_dens_mvee
**24**	Morphology	Area density (MVEE)	morph_area_dens_mvee
**25**	Morphology	Volume density (convex hull)	morph_vol_dens_conv_hull
**26**	Morphology	Area density (convex hull)	morph_area_dens_conv_hull
**27**	Morphology	Integrated intensity	morph_integ_int
**28**	Morphology	Moran's I index	morph_moran_i
**29**	Morphology	Geary's C measure	morph_geary_c
**30**	Local intensity	Local intensity peak	loc_peak_loc
**31**	Local intensity	Global intensity peak	loc_peak_glob
**32**	Statistics	Mean	stat_mean
**33**	Statistics	Variance	stat_var
**34**	Statistics	Skewness	stat_skew
**35**	Statistics	(Excess) kurtosis	stat_kurt
**36**	Statistics	Median	stat_median
**37**	Statistics	Minimum	stat_min
**38**	Statistics	10th percentile	stat_p10
**39**	Statistics	90th percentile	stat_p90
**40**	Statistics	Maximum	stat_max
**41**	Statistics	Interquartile range	stat_iqr
**42**	Statistics	Range	stat_range
**43**	Statistics	Mean absolute deviation	stat_mad
**44**	Statistics	Robust mean absolute deviation	stat_rmad
**45**	Statistics	Median absolute deviation	stat_medad
**46**	Statistics	Coefficient of variation	stat_cov
**47**	Statistics	Quartile coefficient of dispersion	stat_qcod
**48**	Statistics	Energy	stat_energy
**49**	Statistics	Root mean square	stat_rms
**50**	Intensity histogram	Mean	ih_mean
**51**	Intensity histogram	Variance	ih_var

**52**	Intensity histogram	Skewness	ih_skew
**53**	Intensity histogram	Kurtosis	ih_kurt
**54**	Intensity histogram	Median	ih_median
**55**	Intensity histogram	Minimum	ih_min
**56**	Intensity histogram	10th percentile	ih_p10
**57**	Intensity histogram	90th percentile	ih_p90
**58**	Intensity histogram	Maximum	ih_max
**59**	Intensity histogram	Mode	ih_mode
**60**	Intensity histogram	Interquartile range	ih_iqr
**61**	Intensity histogram	Range	ih_range
**62**	Intensity histogram	Mean absolute deviation	ih_mad
**63**	Intensity histogram	Robust mean absolute deviation	ih_rmad
**64**	Intensity histogram	Median absolute deviation	ih_medad
**65**	Intensity histogram	Coefficient of variation	ih_cov
**66**	Intensity histogram	Quartile coefficient of dispersion	ih_qcod
**67**	Intensity histogram	Entropy	ih_entropy
**68**	Intensity histogram	Uniformity	ih_uniformity
**69**	Intensity histogram	Maximum histogram gradient	ih_max_grad
**70**	Intensity histogram	Maximum gradient grey level	ih_max_grad_g
**71**	Intensity histogram	Minimum histogram gradient	ih_min_grad
**72**	Intensity histogram	Minimum gradient grey level	ih_min_grad_g
**73**	Intensity volume histogram	Volume fraction at 10% intensity	ivh_v10
**74**	Intensity volume histogram	Volume fraction at 90% intensity	ivh_v90
**75**	Intensity volume histogram	Intensity at 10% volume	ivh_i10
**76**	Intensity volume histogram	Intensity at 90% volume	ivh_i90
**77**	Intensity volume histogram	Volume fraction difference between 10% and 90% intensity	ivh_diff_v10_v90
**78**	Intensity volume histogram	Intensity difference between 10% and 90% volume	ivh_diff_i10_i90
**79**	Intensity volume histogram	Area under the IVH curve	ivh_auc
**80**	Co-occurrence matrix (3D, averaged)	Joint maximum	cm_joint_max_3D_avg
**81**	Co-occurrence matrix (3D, averaged)	Joint average	cm_joint_avg_3D_avg
**82**	Co-occurrence matrix (3D, averaged)	Joint variance	cm_joint_var_3D_avg
**83**	Co-occurrence matrix (3D, averaged)	Joint entropy	cm_joint_entr_3D_avg
**84**	Co-occurrence matrix (3D, averaged)	Difference average	cm_diff_avg_3D_avg
**85**	Co-occurrence matrix (3D, averaged)	Difference variance	cm_diff_var_3D_avg
**86**	Co-occurrence matrix (3D, averaged)	Difference entropy	cm_diff_entr_3D_avg
**87**	Co-occurrence matrix (3D, averaged)	Sum average	cm_sum_avg_3D_avg
**88**	Co-occurrence matrix (3D, averaged)	Sum variance	cm_sum_var_3D_avg
**89**	Co-occurrence matrix (3D, averaged)	Sum entropy	cm_sum_entr_3D_avg
**90**	Co-occurrence matrix (3D, averaged)	Angular second moment	cm_energy_3D_avg
**91**	Co-occurrence matrix (3D, averaged)	Contrast	cm_contrast_3D_avg
**92**	Co-occurrence matrix (3D, averaged)	Dissimilarity	cm_dissimilarity_3D_avg
**93**	Co-occurrence matrix (3D, averaged)	Inverse difference	cm_inv_diff_3D_avg
**94**	Co-occurrence matrix (3D, averaged)	Inverse difference normalised	cm_inv_diff_norm_3D_avg
**95**	Co-occurrence matrix (3D, averaged)	Inverse difference moment	cm_inv_diff_mom_3D_avg
**96**	Co-occurrence matrix (3D, averaged)	Inverse difference moment normalised	cm_inv_diff_mom_norm_3D_avg
**97**	Co-occurrence matrix (3D, averaged)	Inverse variance	cm_inv_var_3D_avg
**98**	Co-occurrence matrix (3D, averaged)	Correlation	cm_corr_3D_avg
**99**	Co-occurrence matrix (3D, averaged)	Autocorrelation	cm_auto_corr_3D_avg
**100**	Co-occurrence matrix (3D, averaged)	Cluster tendency	cm_clust_tend_3D_avg
**101**	Co-occurrence matrix (3D, averaged)	Cluster shade	cm_clust_shade_3D_avg
**102**	Co-occurrence matrix (3D, averaged)	Cluster prominence	cm_clust_prom_3D_avg
**103**	Co-occurrence matrix (3D, averaged)	Information correlation 1	cm_info_corr1_3D_avg
**104**	Co-occurrence matrix (3D, averaged)	Information correlation 2	cm_info_corr2_3D_avg
**105**	Co-occurrence matrix (3D, merged)	Joint maximum	cm_joint_max_3D_comb
**106**	Co-occurrence matrix (3D, merged)	Joint average	cm_joint_avg_3D_comb
**107**	Co-occurrence matrix (3D, merged)	Joint variance	cm_joint_var_3D_comb
**108**	Co-occurrence matrix (3D, merged)	Joint entropy	cm_joint_entr_3D_comb
**109**	Co-occurrence matrix (3D, merged)	Difference average	cm_diff_avg_3D_comb
**110**	Co-occurrence matrix (3D, merged)	Difference variance	cm_diff_var_3D_comb

**111**	Co-occurrence matrix (3D, merged)	Difference entropy	cm_diff_entr_3D_comb
**112**	Co-occurrence matrix (3D, merged)	Sum average	cm_sum_avg_3D_comb
**113**	Co-occurrence matrix (3D, merged)	Sum variance	cm_sum_var_3D_comb
**114**	Co-occurrence matrix (3D, merged)	Sum entropy	cm_sum_entr_3D_comb
**115**	Co-occurrence matrix (3D, merged)	Angular second moment	cm_energy_3D_comb
**116**	Co-occurrence matrix (3D, merged)	Contrast	cm_contrast_3D_comb
**117**	Co-occurrence matrix (3D, merged)	Dissimilarity	cm_dissimilarity_3D_comb
**118**	Co-occurrence matrix (3D, merged)	Inverse difference	cm_inv_diff_3D_comb
**119**	Co-occurrence matrix (3D, merged)	Inverse difference normalised	cm_inv_diff_norm_3D_comb
**120**	Co-occurrence matrix (3D, merged)	Inverse difference moment	cm_inv_diff_mom_3D_comb
**121**	Co-occurrence matrix (3D, merged)	Inverse difference moment normalised	cm_inv_diff_mom_norm_3D_comb
**122**	Co-occurrence matrix (3D, merged)	Inverse variance	cm_inv_var_3D_comb
**123**	Co-occurrence matrix (3D, merged)	Correlation	cm_corr_3D_comb
**124**	Co-occurrence matrix (3D, merged)	Autocorrelation	cm_auto_corr_3D_comb
**125**	Co-occurrence matrix (3D, merged)	Cluster tendency	cm_clust_tend_3D_comb
**126**	Co-occurrence matrix (3D, merged)	Cluster shade	cm_clust_shade_3D_comb
**127**	Co-occurrence matrix (3D, merged)	Cluster prominence	cm_clust_prom_3D_comb
**128**	Co-occurrence matrix (3D, merged)	Information correlation 1	cm_info_corr1_3D_comb
**129**	Co-occurrence matrix (3D, merged)	Information correlation 2	cm_info_corr2_3D_comb
**130**	Run length matrix (3D, averaged)	Short runs emphasis	rlm_sre_3D_avg
**131**	Run length matrix (3D, averaged)	Long runs emphasis	rlm_lre_3D_avg
**132**	Run length matrix (3D, averaged)	Low grey level run emphasis	rlm_lgre_3D_avg
**133**	Run length matrix (3D, averaged)	High grey level run emphasis	rlm_hgre_3D_avg
**134**	Run length matrix (3D, averaged)	Short run low grey level emphasis	rlm_srlge_3D_avg
**135**	Run length matrix (3D, averaged)	Short run high grey level emphasis	rlm_srhge_3D_avg
**136**	Run length matrix (3D, averaged)	Long run low grey level emphasis	rlm_lrlge_3D_avg
**137**	Run length matrix (3D, averaged)	Long run high grey level emphasis	rlm_lrhge_3D_avg
**138**	Run length matrix (3D, averaged)	Grey level non-uniformity	rlm_glnu_3D_avg
**139**	Run length matrix (3D, averaged)	Grey level non-uniformity normalised	rlm_glnu_norm_3D_avg
**140**	Run length matrix (3D, averaged)	Run length non-uniformity	rlm_rlnu_3D_avg
**141**	Run length matrix (3D, averaged)	Run length non-uniformity normalised	rlm_rlnu_norm_3D_avg
**142**	Run length matrix (3D, averaged)	Run percentage	rlm_r_perc_3D_avg
**143**	Run length matrix (3D, averaged)	Grey level variance	rlm_gl_var_3D_avg
**144**	Run length matrix (3D, averaged)	Run length variance	rlm_rl_var_3D_avg
**145**	Run length matrix (3D, averaged)	Run entropy	rlm_rl_entr_3D_avg
**146**	Run length matrix (3D, merged)	Short runs emphasis	rlm_sre_3D_comb
**147**	Run length matrix (3D, merged)	Long runs emphasis	rlm_lre_3D_comb
**148**	Run length matrix (3D, merged)	Low grey level run emphasis	rlm_lgre_3D_comb
**149**	Run length matrix (3D, merged)	High grey level run emphasis	rlm_hgre_3D_comb
**150**	Run length matrix (3D, merged)	Short run low grey level emphasis	rlm_srlge_3D_comb
**151**	Run length matrix (3D, merged)	Short run high grey level emphasis	rlm_srhge_3D_comb
**152**	Run length matrix (3D, merged)	Long run low grey level emphasis	rlm_lrlge_3D_comb
**153**	Run length matrix (3D, merged)	Long run high grey level emphasis	rlm_lrhge_3D_comb
**154**	Run length matrix (3D, merged)	Grey level non-uniformity	rlm_glnu_3D_comb
**155**	Run length matrix (3D, merged)	Grey level non-uniformity normalised	rlm_glnu_norm_3D_comb
**156**	Run length matrix (3D, merged)	Run length non-uniformity	rlm_rlnu_3D_comb
**157**	Run length matrix (3D, merged)	Run length non-uniformity normalised	rlm_rlnu_norm_3D_comb
**158**	Run length matrix (3D, merged)	Run percentage	rlm_r_perc_3D_comb
**159**	Run length matrix (3D, merged)	Grey level variance	rlm_gl_var_3D_comb
**160**	Run length matrix (3D, merged)	Run length variance	rlm_rl_var_3D_comb
**161**	Run length matrix (3D, merged)	Run entropy	rlm_rl_entr_3D_comb
**162**	Size zone matrix (3D)	Small zone emphasis	szm_sze_3D
**163**	Size zone matrix (3D)	Large zone emphasis	szm_lze_3D
**164**	Size zone matrix (3D)	Low grey level emphasis	szm_lgze_3D
**165**	Size zone matrix (3D)	High grey level emphasis	szm_hgze_3D
**166**	Size zone matrix (3D)	Small zone low grey level emphasis	szm_szlge_3D
**167**	Size zone matrix (3D)	Small zone high grey level emphasis	szm_szhge_3D
**168**	Size zone matrix (3D)	Large zone low grey level emphasis	szm_lzlge_3D
**169**	Size zone matrix (3D)	Large zone high grey level emphasis	szm_lzhge_3D

**170**	Size zone matrix (3D)	Grey level non-uniformity	szm_glnu_3D
**171**	Size zone matrix (3D)	Grey level non uniformity normalised	szm_glnu_norm_3D
**172**	Size zone matrix (3D)	Zone size non-uniformity	szm_zsnu_3D
**173**	Size zone matrix (3D)	Zone size non-uniformity normalised	szm_zsnu_norm_3D
**174**	Size zone matrix (3D)	Zone percentage	szm_z_perc_3D
**175**	Size zone matrix (3D)	Grey level variance	szm_gl_var_3D
**176**	Size zone matrix (3D)	Zone size variance	szm_zs_var_3D
**177**	Size zone matrix (3D)	Zone size entropy	szm_zs_entr_3D
**178**	Distance zone matrix (3D)	Small distance emphasis	dzm_sde_3D
**179**	Distance zone matrix (3D)	Large distance emphasis	dzm_lde_3D
**180**	Distance zone matrix (3D)	Low grey level emphasis	dzm_lgze_3D
**181**	Distance zone matrix (3D)	High grey level emphasis	dzm_hgze_3D
**182**	Distance zone matrix (3D)	Small distance low grey level emphasis	dzm_sdlge_3D
**183**	Distance zone matrix (3D)	Small distance high grey level emphasis	dzm_sdhge_3D
**184**	Distance zone matrix (3D)	Large distance low grey level emphasis	dzm_ldlge_3D
**185**	Distance zone matrix (3D)	Large distance high grey level emphasis	dzm_ldhge_3D
**186**	Distance zone matrix (3D)	Grey level non-uniformity	dzm_glnu_3D
**187**	Distance zone matrix (3D)	Grey level non-uniformity normalised	dzm_glnu_norm_3D
**188**	Distance zone matrix (3D)	Zone distance non-uniformity	dzm_zdnu_3D
**189**	Distance zone matrix (3D)	Zone distance non-uniformity normalised	dzm_zdnu_norm_3D
**190**	Distance zone matrix (3D)	Zone percentage	dzm_z_perc_3D
**191**	Distance zone matrix (3D)	Grey level variance	dzm_gl_var_3D
**192**	Distance zone matrix (3D)	Zone distance variance	dzm_zd_var_3D
**193**	Distance zone matrix (3D)	Zone distance entropy	dzm_zd_entr_3D
**194**	Neighbourhood grey tone difference matrix (3D)	Coarseness	ngt_coarseness_3D
**195**	Neighbourhood grey tone difference matrix (3D)	Contrast	ngt_contrast_3D
**196**	Neighbourhood grey tone difference matrix (3D)	Busyness	ngt_busyness_3D
**197**	Neighbourhood grey tone difference matrix (3D)	Complexity	ngt_complexity_3D
**198**	Neighbourhood grey tone difference matrix (3D)	Strength	ngt_strength_3D
**199**	Neighbouring grey level dependence matrix (3D)	Low dependence emphasis	ngl_lde_3D
**200**	Neighbouring grey level dependence matrix (3D)	High dependence emphasis	ngl_hde_3D
**201**	Neighbouring grey level dependence matrix (3D)	Low grey level count emphasis	ngl_lgce_3D
**202**	Neighbouring grey level dependence matrix (3D)	High grey level count emphasis	ngl_hgce_3D
**203**	Neighbouring grey level dependence matrix (3D)	Low dependence low grey level emphasis	ngl_ldlge_3D
**204**	Neighbouring grey level dependence matrix (3D)	Low dependence high grey level emphasis	ngl_ldhge_3D
**205**	Neighbouring grey level dependence matrix (3D)	High dependence low grey level emphasis	ngl_hdlge_3D
**206**	Neighbouring grey level dependence matrix (3D)	High dependence high grey level emphasis	ngl_hdhge_3D
**207**	Neighbouring grey level dependence matrix (3D)	Grey level non-uniformity	ngl_glnu_3D
**208**	Neighbouring grey level dependence matrix (3D)	Grey level non-uniformity normalised	ngl_glnu_norm_3D
**209**	Neighbouring grey level dependence matrix (3D)	Dependence count non-uniformity	ngl_dcnu_3D
**210**	Neighbouring grey level dependence matrix (3D)	Dependence count non-uniformity normalised	ngl_dcnu_norm_3D
**211**	Neighbouring grey level dependence matrix (3D)	Dependence count percentage	ngl_dc_perc_3D
**212**	Neighbouring grey level dependence matrix (3D)	Grey level variance	ngl_gl_var_3D
**213**	Neighbouring grey level dependence matrix (3D)	Dependence count variance	ngl_dc_var_3D
**214**	Neighbouring grey level dependence matrix (3D)	Dependence count entropy	ngl_dc_entr_3D
**215**	Neighbouring grey level dependence matrix (3D)	Dependence count energy	ngl_dc_energy_3D

Supplementary Table 2. Selected features from each modality (columns) by each feature selection method (rows) for each of the centers. A) CHUM, B) CHUS, C) HMR, D) HGJ, E) HNSCC.

Table 2. A) CHUM

	CT	Dose	Dose-CT	LLRR	WF	WLS	Clinical
C-index	Stat-range<br>T-stage<br>Stat-rms<br>Stat-min<br>Stat-energy<br>Stat-cov<br>Morph_pca_elongation	T_stage<br>morph_pca_elongation<br>morph_av<br>morph_pca_least_axis<br>Primary_Site<br>TNM_group_stage<br>ih_cov<br>ih_min_grad_g	CT_stat_range<br>T_stage<br>CT_stat_min<br>Dose_morph_pca_elongation<br>CT_stat_energy	T_stage<br>morph_pca_elongation<br>szm_zs_var_3D<br>stat_iqr<br>rlm_rl_var_3D_avg<br>morph_av<br>Primary_Site<br>stat_qcod	dzm_glnu_3D<br>T_stage<br>szm_zsnu_3D<br>morph_pca_elongation<br>ngt_strength_3D<br>morph_pca_least_axis<br>morph_av	T_stage<br>morph_geary_c<br>morph_pca_least_axis<br>loc_peak_loc<br>ngt_strength_3D<br>stat_min<br>Primary_Site	T_stage<br>Primary_Site<br>TNM_group_stage<br>Age<br>Sex
MI	Sex<br>Therapy<br>Ih-iqr<br>Stat-range<br>Stat-var<br>Stat-max<br>Morph_com<br>Stat-rms<br>Stat-min<br>Stat-energy	Sex<br>Therapy<br>stat_iqr<br>dzm_sde_3D<br>dzm_zdnu_norm_3D<br>ivh_v90<br>morph_pca_elongation<br>morph_av<br>morph_integ_int<br>morph_volume	Sex<br>Therapy<br>CT_ih_iqr<br>CT_stat_range<br>CT_stat_var<br>Dose_stat_iqr<br>Dose_dzm_sde_3D<br>Dose_dzm_zdnu_norm_3D<br>CT_stat_max<br>CT_morph_com	ih_iqr<br>ih_qcod<br>loc_peak_glob<br>ivh_diff_i10_i90<br>ih_rmad<br>ih_min<br>ih_min_grad_g<br>ivh_diff_v10_v90<br>ih_p90<br>ih_range	ih_p90<br>ivh_i10<br>ih_iqr<br>ih_qcod<br>loc_peak_glob<br>ih_median<br>ih_mode<br>Sex<br>ih_p10<br>ivh_i90	ih_p90<br>ivh_i10<br>ih_median<br>ih_mode<br>loc_peak_glob<br>Sex<br>ih_p10<br>ivh_i90<br>Therapy<br>ih_iqr	Sex<br>Therapy<br>Primary_Site<br>TNM_group_stage<br>Age<br>T_stage<br>N_stage
VH	morph_pca_least_axis<br>ivh_i10<br>morph_area_mesh<br>morph_area_dens_ombb<br>stat_var<br>stat_p90<br>morph_volume<br>morph_vol_dens_aabb<br>morph_area_dens_conv_hull<br>morph_geary_c	ngt_strength_3D<br>morph_av<br>szm_lzhge_3D<br>Age<br>morph_area_mesh<br>morph_diam<br>morph_area_dens_aabb<br>stat_mean<br>stat_p90<br>ngt_coarseness_3D	CT_stat_cov<br>Dose_morph_volume<br>Dose_ngt_strength_3D<br>T_stage<br>CT_morph_com<br>CT_loc_peak_glob<br>Dose_morph_vol_approx<br>Dose_ngl_glnu_3D<br>CT_stat_range<br>Dose_stat_energy	morph_vol_dens_aee<br>morph_area_dens_mvee<br>rlm_rl_var_3D_comb<br>morph_volume<br>Primary_Site<br>T_stage<br>stat_range<br>stat_mad<br>rlm_srhge_3D_avg<br>rlm_rl_var_3D_avg	szm_zsnu_3D<br>szm_zsnu_norm_3D<br>dzm_ldlge_3D<br>Age<br>T_stage<br>stat_min<br>stat_range<br>rlm_rlnu_3D_avg<br>szm_glnu_3D<br>szm_zs_entr_3D	morph_area_dens_aee<br>rlm_rlnu_3D_comb<br>dzm_zdnu_3D<br>morph_integ_int<br>morph_area_mesh<br>stat_range<br>rlm_glnu_3D_comb<br>szm_lgze_3D<br>szm_szhge_3D<br>szm_zsnu_3D	T_stage<br>N_stage
VH.VIMP	Primary_Site<br>T_stage<br>morph_area_mesh<br>morph_pca_elongation<br>stat_var<br>stat_max<br>stat_cov	morph_volume<br>morph_vol_approx<br>morph_pca_elongation<br>szm_glnu_3D<br>dzm_sdlge_3D<br>dzm_glnu_3D	Primary_Site<br>CT_morph_volume<br>CT_stat_range<br>CT_stat_cov<br>Dose_morph_vol_approx<br>Dose_rlm_glnu_3D_comb	Primary_Site<br>TNM_group_stage<br>morph_vol_approx<br>morph_area_mesh<br>morph_integ_int<br>ngl_glnu_3D	Primary_Site<br>morph_volume<br>morph_pca_elongation<br>morph_integ_int<br>loc_peak_loc<br>szm_glnu_3D<br>dzm_glnu_3D	T_stage<br>morph_area_mesh<br>morph_pca_elongation<br>morph_area_dens_aabb<br>dzm_glnu_3D<br>dzm_zdnu_3D	Age<br>Therapy
MD	N_stage<br>Primary_Site<br>stat_cov<br>TNM_group_stage<br>morph_pca_elongation<br>T_stage	N_stage<br>Primary_Site<br>TNM_group_stage<br>morph_pca_elongation<br>Age<br>T_stage<br>morph_volume<br>morph_vol_approx<br>stat_min<br>morph_integ_int	N_stage<br>CT_stat_cov<br>CT_dzm_lde_3D<br>Dose_rlm_glnu_3D_avg<br>Dose_stat_energy<br>TNM_group_stage<br>Dose_rlm_glnu_3D_comb<br>CT_morph_pca_elongation<br>Dose_morph_com<br>CT_morph_com	Primary_Site<br>N_stage<br>TNM_group_stage<br>T_stage<br>Age<br>morph_pca_elongation<br>morph_integ_int<br>morph_area_dens_conv_hull<br>morph_pca_least_axis<br>stat_range	Primary_Site<br>morph_geary_c<br>T_stage<br>Age<br>szm_glnu_3D<br>N_stage<br>szm_zs_entr_3D<br>dzm_glnu_3D<br>morph_pca_elongation<br>dzm_zd_entr_3D	Primary_Site<br>szm_sze_3D<br>N_stage<br>TNM_group_stage<br>T_stage<br>stat_min<br>szm_szhge_3D<br>Age<br>morph_geary_c<br>szm_glnu_3D	T_stage<br>Primary_Site<br>N_stage<br>Age<br>TNM_group_stage<br>Sex<br>Therapy

Table 2*. B) CHUS*

	CT	Dose	Dose-CT	LLRR	WF	WLS	clinical
C-index	stat_range<br>Primary_Site<br>morph_area_dens_conv_hull<br>morph_pca_elongation<br>morph_pca_min_axis	Primary_Site<br>morph_pca_elongation<br>morph_integ_int<br>morph_pca_least_axis<br>ih_p10	CT_stat_range<br>Primary_Site<br>CT_morph_pca_elongation<br>Dose_morph_integ_int<br>CT_morph_integ_int<br>CT_loc_peak_glob<br>CT_morph_pca_min_axis<br>CT_stat_cov	Primary_Site<br>morph_pca_elongation<br>T_stage<br>stat_range<br>morph_pca_least_axis<br>morph_pca_min_axis<br>morph_area_dens_ombb<br>stat_max	Primary_Site<br>morph_pca_elongation<br>dzm_glnu_3D<br>T_stage<br>dzm_zdnu_3D<br>szm_sze_3D<br>morph_pca_least_axis<br>stat_range	Primary_Site<br>morph_area_dens_conv_hull<br>morph_pca_elongation<br>T_stage<br>morph_pca_least_axis<br>morph_pca_min_axis<br>stat_range<br>loc_peak_loc<br>morph_area_dens_ombb	Primary_Site<br>T_stage<br>TNM_group_stage<br>Age<br>Therapy
MI	Sex<br>Therapy<br>ih_iqr<br>stat_range<br>stat_max<br>morph_area_dens_conv_hull<br>morph_pca_elongation<br>ih_min_grad_g<br>ih_mode<br>ih_max_grad_g	Sex<br>Therapy<br>ivh_v90<br>stat_iqr<br>morph_pca_elongation<br>morph_integ_int<br>morph_pca_min_axis<br>morph_volume<br>morph_vol_approx<br>morph_area_mesh	Sex<br>Therapy<br>CT_ih_iqr<br>Dose_ivh_v90<br>CT_stat_range<br>CT_stat_max<br>Dose_stat_iqr<br>CT_morph_area_dens_conv_hull<br>Dose_morph_pca_elongation<br>CT_morph_pca_elongation	ih_iqr<br>ih_qcod<br>ivh_diff_i10_i90<br>ih_rmad<br>loc_peak_glob<br>ih_median<br>ih_p90<br>ih_mode<br>ih_range<br>ih_max_grad_g	ih_p90<br>ivh_i10<br>ih_iqr<br>ih_qcod<br>ih_median<br>ih_mode<br>loc_peak_glob<br>Sex<br>ih_p10<br>ivh_i90	ih_p90<br>ivh_i10<br>ih_median<br>ih_mode<br>Sex<br>Therapy<br>loc_peak_glob<br>ivh_diff_i10_i90<br>ih_p10<br>ivh_i90	Sex<br>Therapy<br>TNM_group_stage<br>Age<br>T_stage<br>N_stage<br>Primary_Site
VH	Primary_Site<br>morph_area_mesh<br>ivh_i10<br>morph_pca_elongation<br>ngt_strength_3D<br>dzm_sde_3D<br>morph_diam<br>morph_geary_c<br>loc_peak_glob<br>dzm_glnu_3D	morph_pca_min_axis<br>TNM_group_stage<br>morph_volume<br>Age<br>morph_vol_approx<br>morph_pca_elongation<br>morph_area_dens_conv_hull<br>morph_integ_int<br>morph_moran_i<br>loc_peak_loc	Dose_morph_volume<br>CT_morph_area_dens_conv_hull<br>CT_ivh_i10<br>CT_rlm_rlnu_3D_avg<br>Dose_morph_integ_int<br>TNM_group_stage<br>Dose_morph_area_dens_conv_hull<br>Dose_ivh_v90<br>CT_morph_pca_least_axis<br>CT_morph_pca_elongation	morph_area_dens_conv_hull<br>szm_zs_entr_3D<br>T_stage<br>morph_area_dens_aabb<br>morph_av<br>morph_area_dens_aee<br>ngl_glnu_3D<br>Age<br>TNM_group_stage<br>morph_volume	morph_area_mesh<br>morph_area_dens_ombb<br>morph_pca_elongation<br>N_stage<br>loc_peak_loc<br>stat_medad<br>rlm_rlnu_3D_comb<br>dzm_sdlge_3D<br>morph_volume<br>morph_integ_int	szm_szlge_3D<br>morph_vol_approx<br>morph_pca_least_axis<br>morph_area_dens_ombb<br>Age<br>cm_corr_3D_avg<br>szm_zsnu_norm_3D<br>morph_area_mesh<br>morph_area_dens_aabb	Primary_Site<br>T_stage
VH.VIMP	TNM_group_stage<br>morph_volume<br>morph_area_mesh<br>morph_pca_least_axis<br>stat_var<br>stat_range<br>stat_cov	TNM_group_stage<br>morph_volume<br>morph_area_dens_conv_hull<br>loc_peak_loc<br>loc_peak_glob<br>stat_mean<br>stat_p10	CT_morph_volume<br>CT_morph_vol_approx<br>CT_morph_area_dens_conv_hull<br>CT_morph_integ_int<br>Dose_morph_vol_approx<br>Dose_morph_area_mesh	morph_volume<br>morph_area_mesh<br>morph_pca_least_axis<br>morph_area_dens_conv_hull<br>morph_integ_int	Primary_Site<br>TNM_group_stage<br>morph_volume<br>morph_vol_approx<br>morph_area_mesh<br>morph_area_dens_aee<br>morph_area_dens_conv_hull	Primary_Site<br>morph_volume<br>morph_vol_approx<br>morph_area_mesh<br>morph_area_dens_aee<br>loc_peak_loc	Primary_Site<br>N_stage
MD	Primary_Site<br>morph_area_dens_conv_hull<br>N_stage<br>morph_vol_approx<br>morph_volume<br>TNM_group_stage<br>ivh_i10<br>morph_pca_least_axis<br>morph_area_mesh<br>Age	Primary_Site<br>N_stage<br>morph_vol_approx<br>TNM_group_stage<br>morph_volume<br>Age<br>morph_integ_int<br>morph_area_mesh<br>morph_pca_least_axis<br>morph_pca_elongation	Primary_Site<br>Dose_morph_integ_int<br>N_stage<br>Dose_morph_volume<br>Dose_morph_vol_approx<br>CT_morph_area_dens_conv_hull<br>Dose_morph_area_mesh<br>CT_morph_pca_least_axis<br>CT_morph_volume<br>CT_morph_vol_approx	Primary_Site<br>morph_integ_int<br>morph_volume<br>N_stage<br>morph_pca_least_axis<br>morph_vol_approx<br>morph_area_dens_conv_hull<br>morph_area_mesh<br>Age<br>TNM_group_stage	Primary_Site<br>morph_area_dens_conv_hull<br>morph_volume<br>stat_range<br>TNM_group_stage<br>stat_min<br>morph_pca_least_axis<br>N_stage<br>morph_vol_approx<br>morph_integ_int	Primary_Site<br>morph_area_dens_conv_hull<br>N_stage<br>TNM_group_stage<br>stat_min<br>Age<br>morph_integ_int<br>szm_sze_3D<br>morph_vol_approx<br>morph_area_mesh	Primary_Site<br>T_stage<br>N_stage<br>TNM_group_stage<br>Age<br>Therapy<br>Sex

Table 2. *C) HMR*

	CT	Dose	Dose-CT	LLRR	WF	WLS	Clinical
C-index	morph_pca_least_axis<br>T_stage<br>stat_range<br>morph_pca_min_axis<br>morph_area_dens_conv_hull<br>Primary_Site	T_stage<br>dzm_zd_entr_3D<br>ivh_v10<br>ih_cov<br>morph_pca_min_axis<br>stat_kurt	T_stage<br>Dose_dzm_zd_entr_3D<br>CT_stat_range<br>Dose_ivh_v10<br>Dose_ih_cov<br>Dose_dzm_glnu_norm_3D<br>Dose_morph_integ_int<br>Dose_ih_rmad	morph_pca_least_axis<br>T_stage<br>rlm_rlnu_3D_avg<br>stat_iqr<br>morph_pca_min_axis<br>stat_range<br>szm_zs_var_3D	T_stage<br>stat_iqr<br>dzm_zdnu_3D<br>morph_integ_int<br>morph_pca_min_axis<br>dzm_glnu_3D<br>szm_zsnu_3D<br>morph_area_dens_conv_hull	morph_pca_least_axis<br>T_stage<br>loc_peak_loc<br>morph_pca_min_axis<br>dzm_z_perc_3D<br>Primary_Site	T_stage<br>Primary_Site<br>TNM_group_stage<br>N_stage<br>Age<br>Sex
MI	Sex<br>Therapy<br>ih_iqr<br>morph_pca_least_axis<br>Primary_Site<br>stat_range<br>morph_area_mesh<br>stat_max<br>morph_vol_approx<br>morph_volume	Sex<br>Therapy<br>morph_pca_least_axis<br>Primary_Site<br>morph_integ_int<br>dzm_zd_entr_3D<br>ivh_v10<br>ih_cov<br>morph_vol_approx<br>morph_volume	Sex<br>Therapy<br>CT_ih_iqr<br>Dose_morph_pca_least_axis<br>CT_morph_pca_least_axis<br>Primary_Site<br>CT_stat_range<br>Dose_morph_integ_int<br>Dose_dzm_zd_entr_3D<br>Dose_ivh_v10	ih_iqr<br>ih_qcod<br>ivh_diff_i10_i90<br>ih_rmad<br>loc_peak_glob<br>ih_p90<br>ih_range<br>ih_max_grad_g<br>ivh_i10<br>ih_median	ih_p90<br>ivh_i10<br>ih_iqr<br>ih_qcod<br>ih_median<br>ih_mode<br>loc_peak_glob<br>Sex<br>Therapy<br>ivh_diff_i10_i90	ih_p90<br>ivh_i10<br>ih_median<br>ih_mode<br>ih_iqr<br>ih_qcod<br>Sex<br>loc_peak_glob<br>ivh_diff_i10_i90<br>Therapy	Sex<br>Therapy<br>Primary_Site<br>TNM_group_stage<br>N_stage<br>T_stage<br>Age
VH	morph_pca_least_axis<br>loc_peak_glob<br>rlm_rlnu_3D_avg<br>morph_area_mesh<br>ivh_i10<br>cm_info_corr2_3D_avg<br>rlm_rlnu_3D_comb<br>szm_glnu_3D<br>dzm_zdnu_3D<br>stat_iqr	morph_integ_int<br>morph_volume<br>morph_pca_min_axis<br>morph_vol_approx<br>morph_area_mesh<br>morph_pca_least_axis<br>ih_cov<br>morph_diam<br>ih_var	CT_morph_area_dens_conv_hull<br>Dose_morph_volume<br>CT_cm_info_corr2_3D_comb<br>CT_szm_glnu_3D<br>CT_morph_area_mesh<br>CT_morph_pca_least_axis<br>CT_morph_area_dens_aee<br>CT_cm_info_corr2_3D_avg<br>CT_ngt_strength_3D<br>Dose_morph_pca_elongation	stat_qcod<br>TNM_group_stage<br>morph_pca_least_axis<br>ngl_glnu_3D<br>morph_vol_dens_aee<br>morph_geary_c<br>rlm_rl_var_3D_avg<br>Primary_Site<br>morph_pca_elongation<br>cm_info_corr1_3D_comb	stat_min<br>morph_area_mesh<br>morph_diam<br>morph_pca_elongation<br>dzm_zdnu_3D<br>Primary_Site<br>rlm_rlnu_3D_comb<br>TNM_group_stage<br>morph_vol_approx<br>szm_zsnu_3D	szm_szhge_3D<br>dzm_zdnu_3D<br>rlm_glnu_3D_comb<br>morph_vol_approx<br>morph_area_dens_aee<br>morph_vol_dens_conv_hull<br>loc_peak_loc<br>cm_info_corr1_3D_avg<br>dzm_zd_entr_3D<br>T_stage	Age<br>Primary_Site
VH.VIMP	Primary_Site<br>morph_vol_approx<br>morph_area_mesh<br>morph_pca_least_axis<br>szm_glnu_3D	Primary_Site<br>morph_volume<br>morph_vol_approx<br>morph_area_mesh<br>morph_integ_int<br>ivh_v10	Primary_Site<br>CT_morph_diam<br>CT_szm_glnu_3D<br>Dose_morph_volume<br>Dose_morph_vol_approx<br>Dose_morph_diam	morph_volume<br>morph_area_mesh<br>morph_integ_int<br>rlm_glnu_3D_avg<br>rlm_glnu_3D_comb	Primary_Site<br>morph_volume<br>morph_vol_approx<br>morph_area_mesh<br>szm_glnu_3D<br>dzm_glnu_3D	Primary_Site<br>morph_vol_approx<br>morph_area_mesh<br>morph_diam<br>morph_integ_int	Age<br>T_stage
MD	Primary_Site<br>morph_geary_c<br>stat_cov<br>TNM_group_stage<br>morph_diam<br>ih_rmad<br>rlm_rlnu_3D_avg<br>morph_area_dens_aee<br>dzm_sdlge_3D<br>rlm_rlnu_3D_comb	Primary_Site<br>morph_area_mesh<br>morph_volume<br>TNM_group_stage<br>morph_diam<br>morph_vol_approx<br>morph_integ_int<br>morph_pca_elongation<br>ivh_diff_v10_v90<br>morph_pca_least_axis	Primary_Site<br>CT_morph_geary_c<br>CT_stat_cov<br>CT_rlm_rlnu_3D_avg<br>CT_rlm_rlnu_3D_comb<br>CT_ih_rmad<br>TNM_group_stage<br>CT_szm_glnu_3D<br>CT_ngt_strength_3D<br>CT_cm_info_corr2_3D_avg	Primary_Site<br>TNM_group_stage<br>morph_area_dens_aee<br>morph_geary_c<br>stat_range<br>morph_area_dens_conv_hull<br>szm_zsnu_norm_3D<br>Age<br>szm_zs_entr_3D<br>rlm_glnu_3D_avg	morph_geary_c<br>Primary_Site<br>szm_zsnu_3D<br>szm_zs_entr_3D<br>stat_range<br>morph_area_dens_aee<br>dzm_zdnu_3D<br>dzm_glnu_3D<br>rlm_rlnu_3D_comb<br>szm_glnu_3D	szm_sze_3D<br>Primary_Site<br>szm_szhge_3D<br>stat_min<br>morph_area_dens_aee<br>TNM_group_stage<br>morph_area_dens_conv_hull<br>morph_geary_c<br>szm_zsnu_norm_3D<br>stat_range	Primary_Site<br>T_stage<br>Age<br>TNM_group_stage<br>N_stage<br>Therapy<br>Sex

Table 2. *D) HGJ*

	CT	Dose	Dose-CT	LLRR	WF	WLS	Clinical
C-index	stat_range<br>T_stage<br>loc_peak_glob<br>TNM_group_stage<br>Primary_Site<br>morph_pca_least_axis<br>morph_pca_min_axis<br>morph_integ_int	morph_integ_int<br>T_stage<br>TNM_group_stage<br>stat_range<br>Primary_Site<br>morph_pca_least_axis<br>ivh_v10	CT_stat_range<br>CT_morph_area_dens_conv_hull<br>TNM_group_stage<br>Primary_Site<br>CT_morph_pca_least_axis<br>Dose_ngl_dc_energy_3D<br>CT_ih_p10	morph_area_dens_conv_hull<br>T_stage<br>TNM_group_stage<br>Primary_Site<br>morph_pca_least_axis<br>morph_pca_min_axis<br>morph_pca_elongation<br>morph_asphericity	T_stage<br>TNM_group_stage<br>Primary_Site<br>morph_pca_least_axis<br>dzm_glnu_3D<br>stat_range<br>szm_zsnu_3D	morph_area_dens_conv_hull<br>T_stage<br>stat_min<br>TNM_group_stage<br>Primary_Site<br>morph_pca_least_axis<br>loc_peak_loc<br>morph_asphericity	T_stage<br>TNM_group_stage<br>Primary_Site<br>Age<br>Sex
MI	Sex<br>Therapy<br>ih_iqr<br>stat_range<br>stat_max<br>ih_min_grad_g<br>ih_p90<br>ih_mode<br>ih_max_grad_g<br>ih_median	Sex<br>Therapy<br>morph_integ_int<br>morph_pca_least_axis<br>morph_pca_min_axis<br>szm_glnu_norm_3D<br>dzm_glnu_norm_3D<br>dzm_zdnu_3D<br>ngl_dc_energy_3D<br>szm_zsnu_3D	Sex<br>Therapy<br>CT_ih_iqr<br>CT_stat_range<br>CT_stat_max<br>CT_ih_min_grad_g<br>CT_ih_p90<br>CT_ih_mode<br>CT_ih_max_grad_g<br>CT_ih_median	ih_iqr<br>ih_qcod<br>loc_peak_glob<br>ivh_diff_i10_i90<br>ih_rmad<br>ih_min<br>ih_min_grad_g<br>ivh_diff_v10_v90<br>ih_p90<br>ih_range	ih_p90<br>ivh_i10<br>ih_iqr<br>ih_qcod<br>ih_median<br>ih_mode<br>loc_peak_glob<br>Sex<br>ih_p10<br>ivh_i90	ih_p90<br>ivh_i10<br>ih_median<br>ih_mode<br>loc_peak_glob<br>Sex<br>ih_p10<br>ivh_i90<br>ivh_diff_i10_i90<br>Therapy	Sex<br>Therapy<br>Primary_Site<br>Age<br>TNM_group_stage<br>N_stage<br>T_stage
VH	morph_area_dens_aee<br>Age<br>stat_max<br>Primary_Site<br>morph_area_dens_conv_hull<br>stat_qcod<br>ih_p90<br>ih_rmad<br>dzm_sde_3D<br>ngl_dcnu_3D	T_stage<br>morph_pca_maj_axis<br>morph_av<br>rlm_rlnu_3D_comb<br>Primary_Site<br>N_stage<br>TNM_group_stage<br>morph_area_mesh<br>morph_area_dens_conv_hull<br>stat_mean	CT_stat_max<br>CT_ivh_i10<br>CT_ih_rmad<br>Dose_ivh_v90<br>TNM_group_stage<br>CT_morph_pca_maj_axis<br>CT_morph_area_dens_conv_hull<br>CT_rlm_rlnu_3D_avg<br>Dose_morph_volume<br>Dose_rlm_rlnu_3D_avg	Age<br>morph_pca_elongation<br>Primary_Site<br>morph_pca_least_axis<br>stat_min<br>szm_zsnu_3D<br>TNM_group_stage<br>rlm_glnu_3D_avg<br>rlm_glnu_3D_comb<br>morph_vol_approx	morph_integ_int<br>morph_geary_c<br>TNM_group_stage<br>morph_volume<br>morph_area_dens_conv_hull<br>morph_area_dens_aee<br>cm_clust_shade_3D_avg<br>rlm_srhge_3D_comb<br>szm_zsnu_3D<br>morph_pca_flatness	morph_volume<br>morph_area_dens_aee<br>N_stage<br>morph_vol_approx<br>morph_pca_elongation<br>rlm_rlnu_3D_comb<br>szm_szhge_3D<br>morph_area_mesh	Therapy<br>Primary_Site
VH.VIMP	Primary_Site<br>TNM_group_stage<br>morph_area_mesh<br>morph_area_dens_ombb<br>morph_area_dens_conv_hull<br>morph_integ_int<br>stat_cov	Primary_Site<br>TNM_group_stage<br>morph_volume<br>morph_pca_least_axis<br>morph_area_dens_conv_hull<br>morph_integ_int<br>rlm_rlnu_3D_avg	CT_morph_area_dens_conv_hull<br>CT_morph_integ_int<br>CT_stat_max<br>CT_stat_cov<br>Dose_morph_volume<br>Dose_morph_vol_approx<br>Dose_morph_area_mesh	Primary_Site<br>TNM_group_stage<br>morph_volume<br>morph_area_dens_ombb<br>morph_area_dens_conv_hull<br>szm_zsnu_norm_3D	morph_asphericity<br>morph_pca_least_axis<br>morph_area_dens_aee<br>morph_area_dens_conv_hull<br>morph_integ_int<br>loc_peak_loc<br>dzm_zdnu_3D	TNM_group_stage<br>morph_volume<br>morph_area_dens_ombb<br>morph_area_dens_conv_hull<br>morph_integ_int<br>stat_min	Sex<br>Age
MD	Primary_Site<br>TNM_group_stage<br>morph_area_dens_conv_hull<br>morph_geary_c<br>Age<br>N_stage<br>ivh_i10<br>szm_glnu_3D<br>stat_median<br>stat_cov	Primary_Site<br>Age<br>TNM_group_stage<br>N_stage<br>ngl_dcnu_norm_3D<br>morph_volume<br>T_stage<br>morph_vol_approx<br>morph_area_dens_conv_hull<br>morph_area_mesh	Primary_Site<br>TNM_group_stage<br>CT_morph_geary_c<br>Age<br>N_stage<br>CT_morph_area_dens_conv_hull<br>CT_stat_cov<br>Dose_morph_area_dens_conv_hull<br>CT_dzm_lde_3D<br>CT_morph_integ_int	Primary_Site<br>morph_area_dens_conv_hull<br>N_stage<br>Age<br>TNM_group_stage<br>morph_integ_int<br>szm_zsnu_norm_3D<br>morph_area_mesh<br>morph_pca_least_axis<br>morph_area_dens_aee	Primary_Site<br>morph_geary_c<br>Age<br>N_stage<br>szm_zsnu_3D<br>stat_range<br>TNM_group_stage<br>morph_area_dens_aee<br>morph_area_dens_conv_hull<br>szm_zs_entr_3D	Primary_Site<br>morph_area_dens_conv_hull<br>szm_sze_3D<br>N_stage<br>Age<br>TNM_group_stage<br>szm_szhge_3D<br>morph_area_mesh<br>morph_area_dens_aee<br>morph_vol_approx	Primary_Site<br>Age<br>TNM_group_stage<br>N_stage<br>T_stage

Table 2 E) HNSCC

	CT	Dose	Dose-CT	LLRR	WF	WLS	Clinical
C-index	TNM_group_stage<br>stat_range<br>Primary_Site<br>T_stage<br>N_stage<br>morph_pca_elongation<br>morph_pca_min_axis<br>morph_area_dens_conv_hull<br>Age<br>stat_median	TNM_group_stage<br>Primary_Site<br>stat_range<br>T_stage<br>dzm_zd_entr_3D<br>stat_min<br>morph_pca_elongation<br>morph_pca_min_axis<br>szm_zsnu_3D	TNM_group_stage<br>CT_stat_range<br>Primary_Site<br>T_stage<br>N_stage<br>Dose_dzm_zd_entr_3D<br>Dose_stat_min	TNM_group_stage<br>Primary_Site<br>T_stage<br>N_stage<br>stat_range<br>stat_max<br>stat_iqr	TNM_group_stage<br>dzm_sdlge_3D<br>Primary_Site<br>T_stage<br>N_stage<br>stat_range<br>morph_pca_elongation<br>stat_iqr	TNM_group_stage<br>loc_peak_loc<br>Primary_Site<br>T_stage<br>N_stage<br>morph_pca_min_axis<br>morph_area_dens_conv_hull<br>Age<br>morph_pca_least_axis	TNM_group_stage<br>Primary_Site<br>N_stage<br>Age<br>Therapy
MI	Therapy<br>Sex<br>ih_iqr<br>stat_range<br>stat_max<br>stat_median<br>morph_pca_min_axis<br>morph_pca_elongation<br>ih_min_grad_g<br>ih_p90	Therapy<br>Sex<br>loc_peak_loc<br>loc_peak_glob<br>stat_mean<br>stat_rms<br>szm_glnu_norm_3D<br>dzm_glnu_norm_3D<br>morph_integ_int<br>stat_p10	Therapy<br>Sex<br>CT_ih_iqr<br>Dose_loc_peak_loc<br>CT_stat_range<br>Dose_loc_peak_glob<br>Dose_stat_mean<br>Dose_stat_rms<br>CT_stat_max<br>Dose_szm_glnu_norm_3D	ih_iqr<br>ih_qcod<br>loc_peak_glob<br>ivh_diff_i10_i90<br>ih_rmad<br>ih_min<br>ih_min_grad_g<br>ivh_diff_v10_v90<br>ih_p90<br>ih_range	ih_p90<br>ivh_i10<br>ih_iqr<br>ih_qcod<br>ih_median<br>ih_mode<br>Therapy<br>loc_peak_glob<br>Sex<br>ih_p10	ih_p90<br>ivh_i10<br>ih_median<br>ih_mode<br>Therapy<br>loc_peak_glob<br>ih_iqr<br>ih_qcod<br>ivh_diff_i10_i90<br>Sex	Therapy<br>Sex<br>Age<br>N_stage<br>Primary_Site<br>TNM_group_stage<br>T_stage
VH	Primary_Site<br>TNM_group_stage<br>stat_max<br>dzm_lde_3D<br>stat_mean<br>stat_median<br>morph_pca_least_axis<br>stat_iqr<br>dzm_ldhge_3D<br>dzm_zd_var_3D	morph_area_mesh<br>morph_integ_int<br>ivh_i90<br>stat_mean<br>ivh_v90<br>Primary_Site<br>morph_com<br>stat_min<br>stat_rms<br>cm_joint_entr_3D_comb	Dose_stat_rms<br>Primary_Site<br>N_stage<br>CT_morph_integ_int<br>Dose_morph_vol_approx<br>Dose_morph_area_mesh<br>Dose_loc_peak_loc<br>Dose_stat_median<br>Dose_stat_max<br>CT_morph_volume	N_stage<br>rlm_rlnu_3D_avg<br>morph_diam<br>morph_volume<br>morph_integ_int<br>dzm_glnu_3D<br>morph_area_mesh<br>morph_pca_maj_axis<br>morph_pca_min_axis<br>morph_area_dens_conv_hull	Age<br>T_stage<br>cm_clust_shade_3D_avg<br>morph_pca_min_axis<br>morph_pca_elongation<br>dzm_zdnu_3D<br>Primary_Site<br>morph_pca_least_axis<br>morph_area_dens_ombb<br>morph_area_dens_conv_hull	szm_sze_3D<br>cm_clust_shade_3D_avg<br>Age<br>Primary_Site<br>morph_vol_approx<br>morph_pca_elongation<br>loc_peak_loc<br>dzm_zdnu_3D<br>morph_area_dens_conv_hull	Primary_Site<br>Therapy
VH.VIMP	Age<br>Primary_Site<br>morph_vol_approx<br>morph_area_mesh<br>cm_clust_prom_3D_avg	Primary_Site<br>morph_area_mesh<br>loc_peak_loc<br>stat_min<br>stat_max<br>stat_rms<br>ivh_i90	CT_morph_integ_int<br>Dose_morph_vol_approx<br>Dose_morph_integ_int<br>Dose_loc_peak_glob<br>Dose_stat_mean<br>Dose_stat_p10	morph_volume<br>morph_vol_approx<br>morph_area_mesh<br>morph_pca_least_axis<br>morph_pca_elongation<br>morph_integ_int	TNM_group_stage<br>loc_peak_loc<br>rlm_lgre_3D_comb<br>rlm_glnu_norm_3D_comb<br>rlm_gl_var_3D_comb<br>dzm_sdlge_3D	TNM_group_stage<br>morph_pca_elongation<br>morph_area_dens_conv_hull<br>rlm_gl_var_3D_comb<br>szm_szhge_3D	Age<br>N_stage
MD	Primary_Site<br>TNM_group_stage<br>N_stage<br>stat_median<br>stat_cov<br>morph_geary_c<br>Age<br>morph_volume<br>morph_vol_approx<br>morph_integ_int	Primary_Site<br>N_stage<br>TNM_group_stage<br>morph_volume<br>morph_integ_int<br>morph_vol_approx<br>ivh_v90<br>morph_area_mesh<br>morph_pca_least_axis<br>stat_mean	Primary_Site<br>N_stage<br>TNM_group_stage<br>Dose_stat_p10<br>CT_morph_geary_c<br>Dose_ivh_i90<br>Dose_ivh_v90<br>CT_stat_cov<br>Dose_stat_mean<br>Dose_morph_area_mesh	Primary_Site<br>TNM_group_stage<br>N_stage<br>stat_range<br>Age<br>morph_integ_int<br>morph_area_mesh<br>morph_pca_least_axis<br>szm_szhge_3D<br>morph_volume	Primary_Site<br>N_stage<br>stat_range<br>loc_peak_loc<br>morph_geary_c<br>TNM_group_stage<br>Age<br>morph_area_mesh<br>morph_integ_int<br>morph_pca_least_axis	Primary_Site<br>TNM_group_stage<br>N_stage<br>szm_sze_3D<br>szm_szhge_3D<br>loc_peak_loc<br>morph_volume<br>morph_area_mesh<br>morph_integ_int<br>morph_vol_approx	Primary_Site<br>Age<br>N_stage<br>TNM_group_stage<br>T_stage<br>Sex

Supplementary Table 3. Machine learning methods, corresponding packages and hyperparameters used in this study.

MODEL	R PACKAGE	HYPERPARAMETERS: RANGE
COXPH	Survival	_
COXBOOST	CoxBoost	maxstepno: 50-500
RSF	randomForestSRC	ntree: 100, 500, 1000<br>mtry:1-10<br>node size: 1:20<br>splitrule: log-rank, logrankscore
GLMBOOST	mboost	mstop: 50-500
GLMNET	glmnet	S: 0.001-0.1<br>alpha: 0-1
ST	rpat	minsplit: 1-20<br>maxdepth: 1-30

Supplementary Table 4. Mean and SD of the C-index for each strategy amongst the different models and related feature selection methods.

	CT	DOSE	Dual_CT_Dose	LLRR	Wavelet	WLS	Clinical
**Coxph_Cindex**	0.66 ± 0.11	0.72 ± 0.14	0.66 ± 0.1	0.67 ± 0.12	0.67 ± 0.12	0.66 ± 0.11	0.69 ± 0.11
**CoxBoost_Cindex**	0.66 ± 0.11	0.7 ± 0.13	0.65 ± 0.11	0.69 ± 0.13	0.66 ± 0.12	0.66 ± 0.11	0.67 ± 0.11
**glmnet_Cindex**	0.68 ± 0.12	0.72 ± 0.14	0.66 ± 0.11	0.69 ± 0.13	0.67 ± 0.12	0.66 ± 0.11	0.66 ± 0.11
**RSF_Cindex**	0.68 ± 0.11	0.67 ± 0.11	0.65 ± 0.095	0.66 ± 0.11	0.64 ± 0.12	0.68 ± 0.11	0.69 ± 0.13
**glmboost_Cindex**	0.67 ± 0.11	0.71 ± 0.13	0.66 ± 0.12	0.69 ± 0.12	0.67 ± 0.12	0.66 ± 0.11	0.65 ± 0.11
**ST_Cindex**	0.63 ± 0.097	0.68 ± 0.12	0.61 ± 0.082	0.61 ± 0.09	0.63 ± 0.1	0.62 ± 0.085	0.65 ± 0.11
**Coxph_MD**	0.65 ± 0.099	0.71 ± 0.12	0.64 ± 0.11	0.72 ± 0.15	0.67 ± 0.1	0.67 ± 0.11	0.68 ± 0.11
**CoxBoost_MD**	0.65 ± 0.1	0.7 ± 0.13	0.61 ± 0.092	0.73 ± 0.15	0.68 ± 0.11	0.67 ± 0.13	0.68 ± 0.11
**glmnet_MD**	0.64 ± 0.099	0.71 ± 0.13	0.62 ± 0.1	0.72 ± 0.15	0.66 ± 0.11	0.66 ± 0.13	0.67 ± 0.11
**RSF_MD**	0.63 ± 0.092	0.71 ± 0.11	0.63 ± 0.091	0.69 ± 0.12	0.64 ± 0.098	0.64 ± 0.098	0.69 ± 0.12
**glmboost_MD**	0.66 ± 0.12	0.7 ± 0.13	0.62 ± 0.091	0.72 ± 0.15	0.67 ± 0.1	0.67 ± 0.12	0.67 ± 0.11
**ST_MD**	0.61 ± 0.096	0.66 ± 0.12	0.61 ± 0.093	0.63 ± 0.099	0.65 ± 0.11	0.61 ± 0.083	0.66 ± 0.12
**Coxph_MI**	0.65 ± 0.11	0.68 ± 0.13	0.62 ± 0.09	0.56 ± 0.055	0.62 ± 0.084	0.6 ± 0.067	0.68 ± 0.11
**CoxBoost_MI**	0.63 ± 0.09	0.68 ± 0.13	0.62 ± 0.086	0.53 ± 0.04	0.6 ± 0.076	0.53 ± 0.049	0.67 ± 0.11
**glmnet_MI**	0.62 ± 0.089	0.68 ± 0.13	0.62 ± 0.09	0.54 ± 0.061	0.6 ± 0.079	0.54 ± 0.057	0.67 ± 0.11
**RSF_MI**	0.65 ± 0.1	0.66 ± 0.12	0.62 ± 0.091	0.59 ± 0.073	0.61 ± 0.085	0.6 ± 0.076	0.71 ± 0.13
**glmboost_MI**	0.63 ± 0.091	0.68 ± 0.13	0.63 ± 0.093	0.53 ± 0.039	0.61 ± 0.078	0.6 ± 0.056	0.67 ± 0.12
**ST_MI**	0.59 ± 0.072	0.59 ± 0.088	0.6 ± 0.082	0.52 ± 0.035	0.57 ± 0.069	0.56 ± 0.045	0.66 ± 0.12
**Coxph_VH.VIMP**	0.71 ± 0.11	0.68 ± 0.12	0.65 ± 0.11	0.7 ± 0.12	0.69 ± 0.12	0.61 ± 0.09	0.65 ± 0.1
**CoxBoost_VH.VIMP**	0.7 ± 0.12	0.71 ± 0.12	0.62 ± 0.094	0.68 ± 0.12	0.68 ± 0.12	0.64 ± 0.11	0.65 ± 0.1
**glmnet_VH.VIMP**	0.7 ± 0.11	0.68 ± 0.12	0.65 ± 0.11	0.69 ± 0.12	0.68 ± 0.12	0.63 ± 0.097	0.66 ± 0.1
**RSF_VH.VIMP**	0.7 ± 0.11	0.66 ± 0.12	0.63 ± 0.087	0.64 ± 0.1	0.67 ± 0.1	0.66 ± 0.097	0.64 ± 0.11
**glmboost_VH.VIMP**	0.69 ± 0.12	0.7 ± 0.13	0.64 ± 0.11	0.7 ± 0.13	0.68 ± 0.12	0.65 ± 0.11	0.65 ± 0.1
**ST_VH.VIMP**	0.63 ± 0.091	0.66 ± 0.14	0.65 ± 0.13	0.66 ± 0.11	0.63 ± 0.09	0.63 ± 0.094	0.63 ± 0.092
**Coxph_VH**	0.65 ± 0.1	0.65 ± 0.11	0.61 ± 0.089	0.72 ± 0.15	0.68 ± 0.099	0.63 ± 0.1	0.64 ± 0.1
**CoxBoost_VH**	0.63 ± 0.1	0.66 ± 0.13	0.62 ± 0.082	0.71 ± 0.14	0.66 ± 0.1	0.65 ± 0.1	0.62 ± 0.099
**glmnet_VH**	0.64 ± 0.11	0.66 ± 0.12	0.61 ± 0.085	0.73 ± 0.15	0.66 ± 0.098	0.62 ± 0.12	0.63 ± 0.1
**RSF_VH**	0.66 ± 0.12	0.66 ± 0.11	0.61 ± 0.081	0.67 ± 0.11	0.68 ± 0.11	0.67 ± 0.11	0.66 ± 0.094
**glmboost_VH**	0.63 ± 0.1	0.66 ± 0.13	0.62 ± 0.084	0.72 ± 0.14	0.67 ± 0.1	0.64 ± 0.099	0.63 ± 0.1
**ST_VH**	0.64 ± 0.11	0.64 ± 0.11	0.62 ± 0.088	0.66 ± 0.13	0.63 ± 0.09	0.64 ± 0.091	0.64 ± 0.086

Supplementary Teble 5-A). The results of Nemenyi post-hoc test showed significant difference among strategies. B) among different feature selection methods, and C) the combination of model and feature selection methods.

	CT	Clinic	DOSE	Dual_CT_Dose	LLRR	WLS	Wavelet
**CT**	1	<0.001	<0.001	<0.001	<0.001	<0.001	<0.001
**Clinic**	<0.001	1	<0.001	<0.001	<0.001	<0.001	<0.001
**DOSE**	<0.001	<0.001	1	<0.001	<0.001	<0.001	<0.001
**Dual_CT_Dose**	<0.001	<0.001	<0.001	1	<0.001	0.99986937	<0.001
**LLRR**	<0.001	<0.001	<0.001	<0.001	1	<0.001	<0.001
**WLS**	<0.001	<0.001	<0.001	0.99986937	<0.001	1	<0.001
**Wavelet**	<0.001	<0.001	<0.001	<0.001	<0.001	<0.001	1

Supplementary Table 5-B). The results of Nemenyi post-hoc test showed significant difference among different feature selection methods

	CoxBoost	Coxph	RSF	ST	glmboost	glmnet
**CoxBoost**	1	<0.001	<0.001	<0.001	<0.001	0.999973
**Coxph**	<0.001	1	<0.001	<0.001	<0.001	<0.001
**RSF**	<0.001	<0.001	1	<0.001	0.998154	<0.001
**ST**	0	<0.001	<0.001	<0.001	<0.001	<0.001
**glmboost**	<0.001	<0.001	0.998154	<0.001	1	<0.001
**glmnet**	0.999973	<0.001	<0.001	<0.001	<0.001	1

Supplementary Table 5-C). The results of Nemenyi post-hoc test showed significant difference among the combination of model and feature selection methods.

	C-index-CoxBoost	C-Index-Coxph	C-index-RSF	C-index-ST	C-index-Glmboost	C-index-Glmnet	MD-CoxBoost	MD-Coxph	MD-RSF	MD-ST	MD-Glmboost	MD-Glmnet	MI-CoxBoost	MI-Coxph	MI-RSF	MI-ST	MI-Glmboost	MI-Glmnet	VH.VIMP-CoxBoost	VH.VIMP-Coxph	VH.VIMP-RSF	VH.VIMP-ST	VH.VIMP-Glmboost	VH.VIMP-Glmnet	VH-CoxBoost	VH-Coxph	VH-RSF	VH-ST	VH-Glmboost	VH-Glmnet
**Cindex Cox Boost**	1	0.01	1	*	1	0.75	1	*	*	*	1	0.91	*	*	*	*	*	*	0.99	1	*	*	1	1	*	*	*	*	*	*
**Cindex Coxph**	0.01	1	*	*	0.08	1	*	1	*	*	0.20	*	*	*	*	*	*	*	*	0.59	*	*	1	0.19	*	*	*	*	*	*
**Cindex RSF**	1	*	1	*	1	*	1	*	0.35	*	1	1	*	*	*	*	*	*	1	0.95	0.01	*	0.26	1	*	*	*	*	*	*
**Cindex ST**	*	*	*	1	*	*	*	*	*	1	*	*	*	0.95	1	*	*	*	*	*	*	*	*	*	*	*	*	*	*	*
**Cindex Glm boost**	1	0.08	1	*	1	0.94	1	0.01	*	*	1	0.68	*	*	*	*	*	*	0.91	1	*	*	1	1	*	*	*	*	*	*
**Cindex Glm net**	0.75	1	*	*	0.94	1	0.49	1	*	*	0.99	*	*	*	*	*	*	*	*	1	*	*	1	0.99	*	*	*	*	*	*
**MD Cox Boost**	1	*	1	*	1	0.49	1	*	*	*	1	0.98	*	*	*	*	*	*	1	1	*	*	0.99	1	*	*	*	*	*	*
**MD Coxph**	*	1	*	*	0.01	1	*	1	*	*	0.02	*	*	*	*	*	*	*	*	0.18	*	*	0.91	0.02	*	*	*	*	*	*
**MD RSF**	*	*	0.35	*	*	*	*	*	1	*	*	1	*	*	*	*	*	*	0.95	*	1	*	*	*	*	*	1	*	*	*
**MD ST**	*	*	*	1	*	*	*	*	*	1	*	*	*	1	1	*	*	*	*	*	*	*	*	*	*	*	*	*	*	*
**MD Glm boost**	1	0.20	1	*	1	0.99	1	0.02	*	*	1	0.46	*	*	*	*	*	*	0.78	1	*	*	1	1	*	*	*	*	*	*
**MD Glm net**	0.91	*	1	*	0.68	*	0.98	*	1	*	0.46	1	*	*	*	*	*	*	1	0.12	0.69	*	*	0.47	*	*	0.06	*	*	*
**MI Cox Boost**	*	*	*	*	*	*	*	*	*	*	*	*	1	*	*	*	*	1	*	*	*	*	*	*	*	*	*	*	*	*
**MI Cox ph**	*	*	*	0.95	*	*	*	*	*	1	*	*	*	1	1	*	*	*	*	*	*	*	*	*	*	*	*	*	*	*
**MI RSF**	*	*	*	1	*	*	*	*	*	1	*	*	*	1	1	*	*	*	*	*	*	*	*	*	*	*	*	*	*	*
**MI ST**	*	*	*	*	*	*	*	*	*	*	*	*	*	*	*	1	*	*	*	*	*	*	*	*	*	*	*	*	*	*
**MI Glm boost**	*	*	*	*	*	*	*	*	*	*	*	*	*	*	*	*	1	*	*	*	*	*	*	*	*	*	*	*	*	*
**MI Glm net**	*	*	*	*	*	*	*	*	*	*	*	*	1	*	*	*	*	1	*	*	*	*	*	*	*	*	*	*	*	*
**VH. VIMP Cox Boost**	0.99	*	1	*	0.91	*	1	*	0.95	*	0.78	1	*	*	*	*	*	*	1	0.36	*	*	0.01	0.78	*	*	0.01	*	*	*
**VH. VIMP Cox ph**	1	0.59	0.95	*	1	1	1	0.18	*	*	1	0.12	*	*	*	*	*	*	0.36	1	*	*	1	1	*	*	*	*	*	*
**VH. VIMP RSF**	*	*	0.01	*	*	*	*	*	1	*	*	0.69	*	*	*	*	*	*	0.36	*	1	*	*	*	*	*	1	*	*	*
**VH. VIMP ST**	*	*	*	*	*	*	*	*	*	*	*	*	*	*	*	*	*	*	*	*	*	1	*	*	*	*	*	1	*	0.33
**VH. VIMP Glm boost**	1	1	0.26	*	1	1	0.99	0.91	*	*	1	*	*	*	*	*	*	*	0.01	1	*	*	1	1	*	*	*	*	*	*

VH. VIMP Glm net	1	0.19	1	*	1	0.99	1	0.02	*	*	1	0.47	*	*	*	*	*	*	0.78	1	*	*	1	1	*	*	*	*	*	*
VH Cox Boost	*	*	*	*	*	*	*	*	*	*	*	*	*	*	*	*	*	*	*	*	*	*	*	*	1	1	*	*	1	0.08
VH Coxph	*	*	*	*	*	*	*	*	*	*	*	*	*	*	*	*	*	*	*	*	*	*	*	*	1	1	*	*	1	*
VH RSF	*	*	*	*	*	*	*	*	1	*	*	0.06	*	*	*	*	*	*	0.01	*	1	*	*	*	*	*	1	*	*	*
VH ST	*	*	*	*	*	*	*	*	*	*	*	*	*	*	*	*	*	*	*	*	*	1	*	*	*	*	*	1	*	0.01
VH Glm boost	*	*	*	*	*	*	*	*	*	*	*	*	*	*	*	*	*	*	*	*	*	*	*	*	1	1	*	*	1	*
VH Glm net	*	*	*	*	*	*	*	*	*	*	*	*	*	*	*	*	*	*	*	*	*	0.33	*	*	0.08	*	*	0.01	*	1

Supplementary Table 6. Top 10% mean of C-index ± SD of different models for all strategies.

Machine	CT	Dose	Dual	LLRR	wavelet	WLS		Clinical
**Coxph-VH. VIMP**	0.71 ± 0.11			0.7±0.12	0.69±0.12			
**CoxBoost-VH. VIMP**	0.7±0.12	0.71±0.12			0.68±0.12			
**glmnet-VH. VIMP**	0.7±0.11				0.68±0.12			
**RSF-VH. VIMP**	0.7±0.11							
**glmBoost-VH-VIMP**		0.7±0.13		0.7±0.13	0.68±0.12			
**CoxPH-Cindex**		0.72±0.14	0.66±0.1					0.69 ± 0.11
**Glmnet-Cindex**		0.72±0.14	066±0.11					
**glmBoost-Cindex**		0.71±0.13	0.66±0.12					
**coxBoost-Cindex**		0.7±0.13						
**RSF-Cindex**						0.68±0.11		0.69 ± 0.13
**Coxph-MD**		0.71±0.12		0.72±0.15		0.67±0.11		
**coxBoost-MD**		0.7±0.13		073±0.15	0.68±0.11	0.67±0.13		
**RSF-MD**		0.71±0.11						0.69 ± 0.12
**Glmnet-MD**		0.71±0.13		0.72±0.15				
**GlmBoost_MD**		0.7±0.13		0.72±0.15		0.67±0.12		
**Glmnet-VH**				0.73±0.15				
**coxPH-VH**				0.72±0.15	0.68±0.0.09			
**CoxBoost-VH**				0.71±0.14				
**glmBoost-VH**				0.7±0.13				
**RSF-VH**					0.68±0.11	0.67±0.11		
**MI_RSF**								0.71 ± 0.13

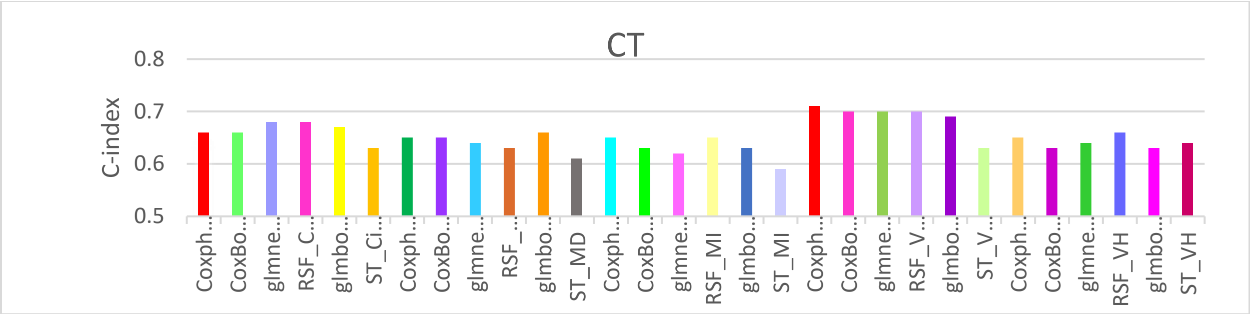
CT
C-index
0.8
0.7
0.6
0.5
Coxph…
CoxBo…
glmne…
RSF_C…
glmbo…
ST_Ci…
Coxph…
CoxBo…
glmne…
RSF_…
glmbo…
ST_MD
Coxph…
CoxBo…
glmne…
RSF_MI
glmbo…
ST_MI
Coxph…
CoxBo…
glmne…
RSF_V…
glmbo…
ST_V…
Coxph…
CoxBo…
glmne…
RSF_VH
glmbo…
ST_VH

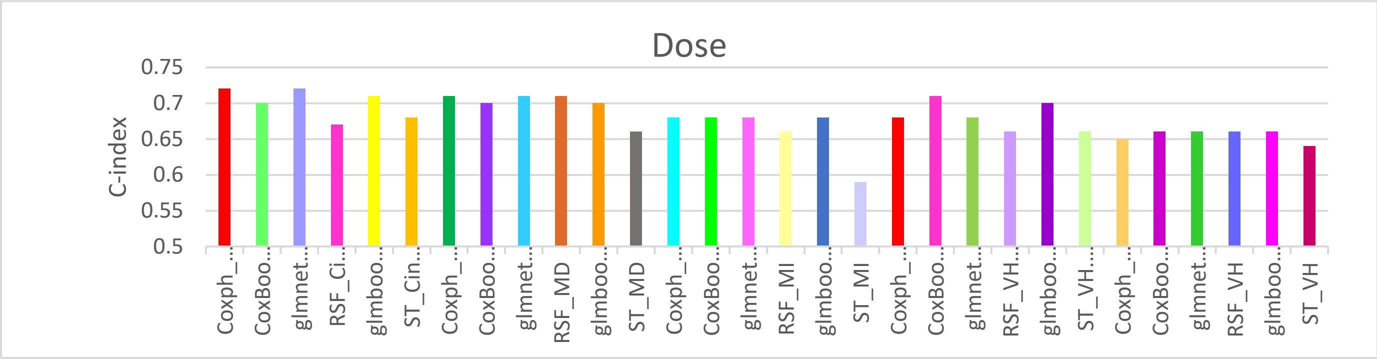
Dose
C-index
0.75
0.7
0.65
0.6
0.55
0.5
Coxph_…
CoxBoo…
glmnet…
RSF_Ci…
glmboo…
ST_Cin…
Coxph_…
CoxBoo…
glmnet…
RSF_MD
glmboo…
ST_MD
Coxph_…
CoxBoo…
glmnet…
RSF_MI
glmboo…
ST_MI
Coxph_…
CoxBoo…
glmnet…
RSF_VH…
glmboo…
ST_VH…
Coxph_…
CoxBoo…
glmnet…
RSF_VH
glmboo…
ST_VH

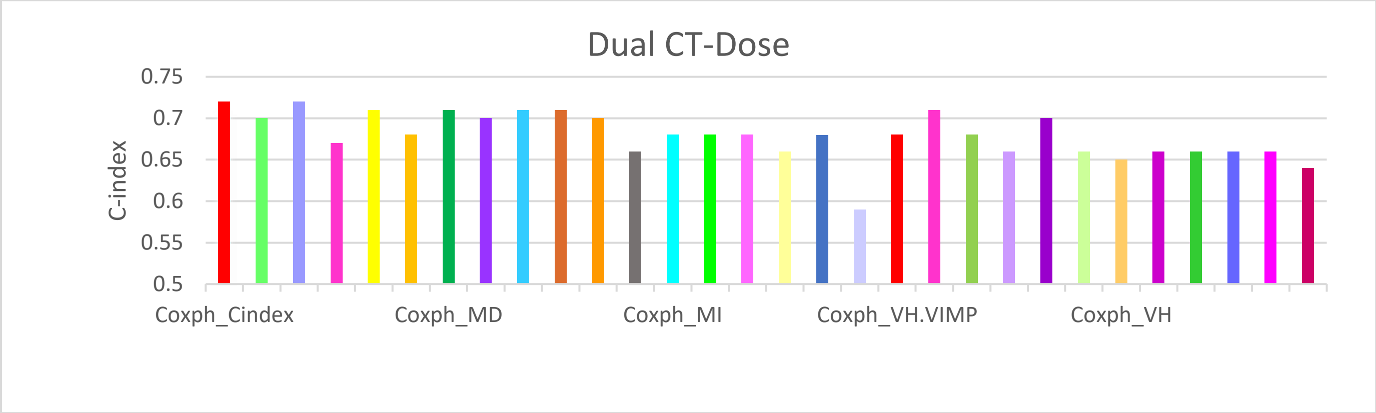
Dual CT-Dose
C-index
0.75
0.7
0.65
0.6
0.55
0.5
Coxph_Cindex
Coxph_MD
Coxph_MI
Coxph_VH.VIMP
Coxph_VH

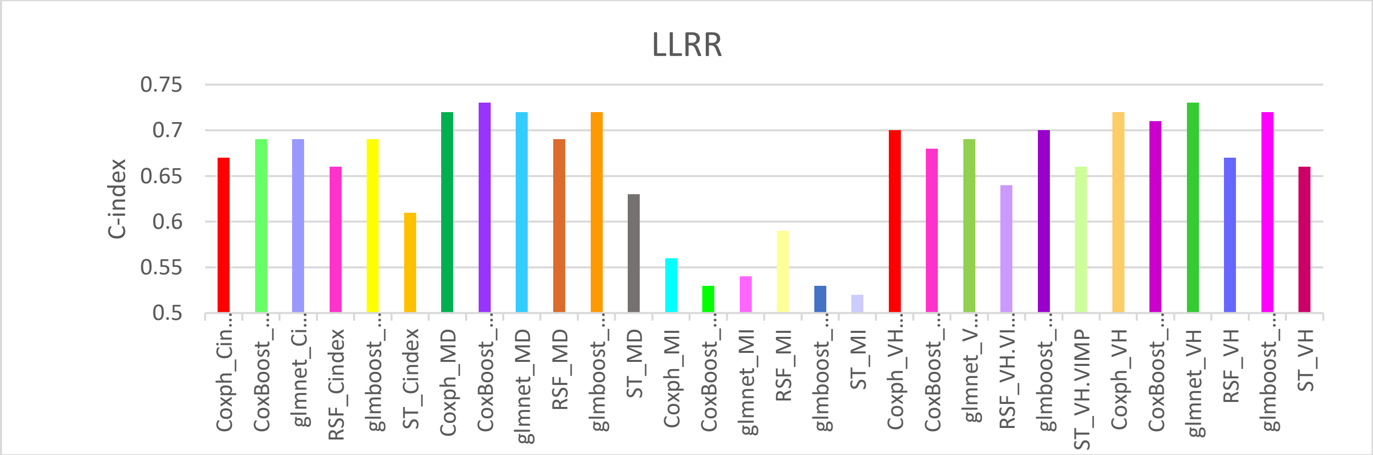
LLRR
C-index
0.75
0.7
0.65
0.6
0.55
0.5
Coxph_Cin…
CoxBoost_…
glmnet_Ci…
RSF_Cindex
glmboost_…
ST_Cindex
Coxph_MD
CoxBoost_…
glmnet_MD
RSF_MD
glmboost_…
ST_MD
Coxph_MI
CoxBoost_…
glmnet_MI
RSF_MI
glmboost_…
ST_MI
Coxph_VH…
CoxBoost_…
glmnet_V…
RSF_VH.VI…
glmboost_…
ST_VH.VIMP
Coxph_VH
CoxBoost_…
glmnet_VH
RSF_VH
glmboost_…
ST_VH

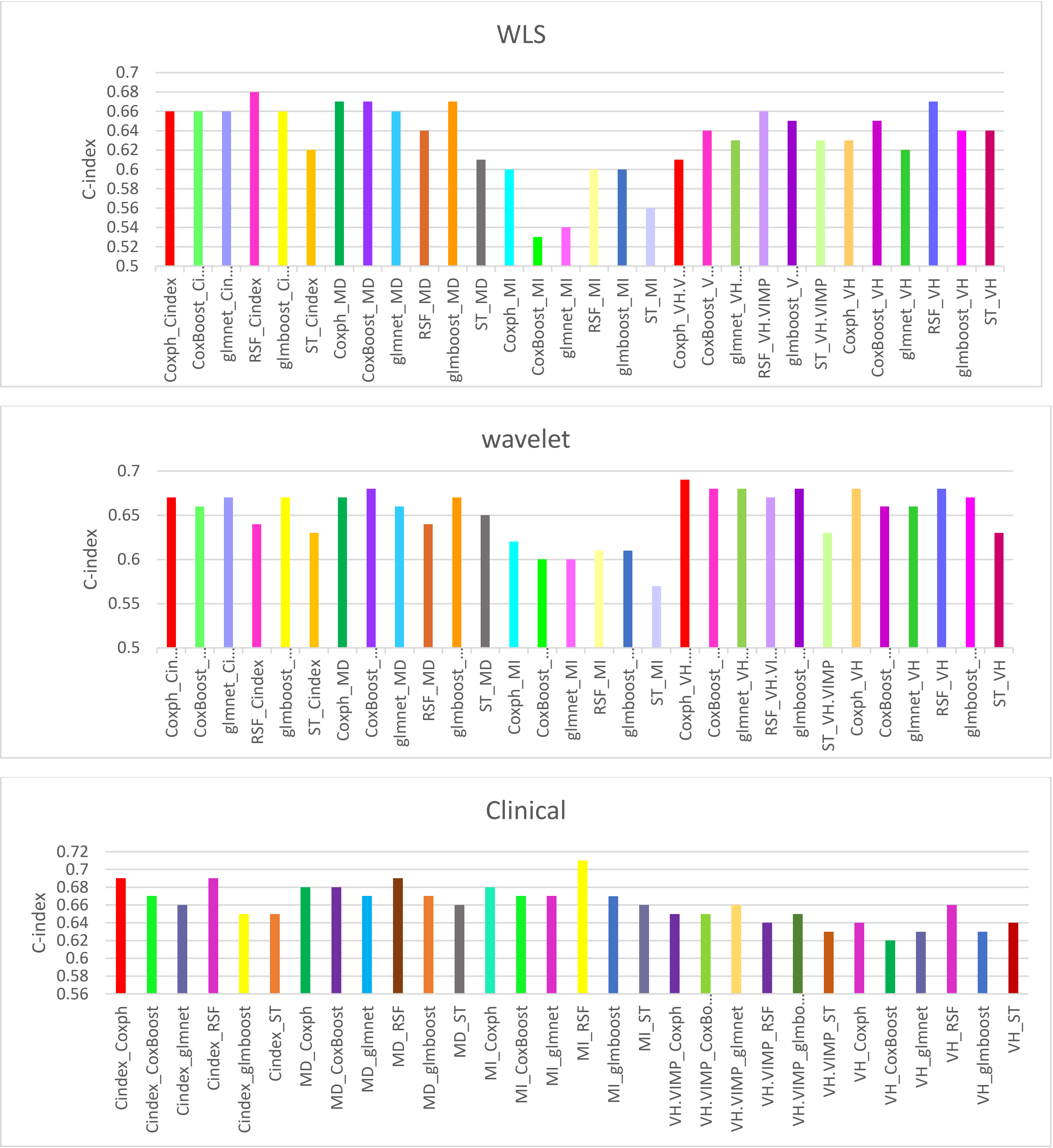

Supplementary figure 1. Detailed bar plots of all C-index achieved separated by the input image selection and machine selection strategies.

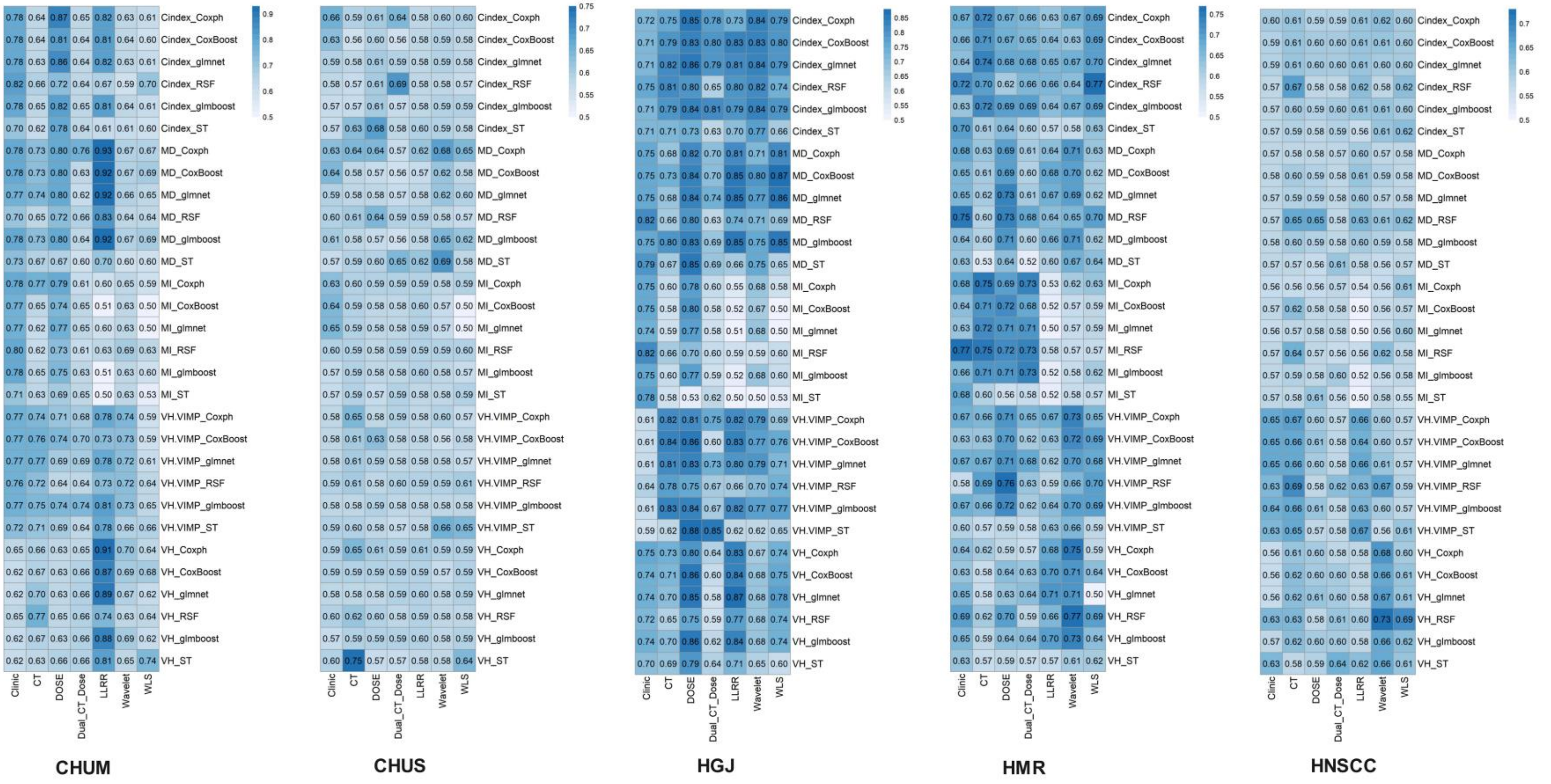

Supplementary figure 2. Heat map of achieved C-indices on a center-by-center basis.

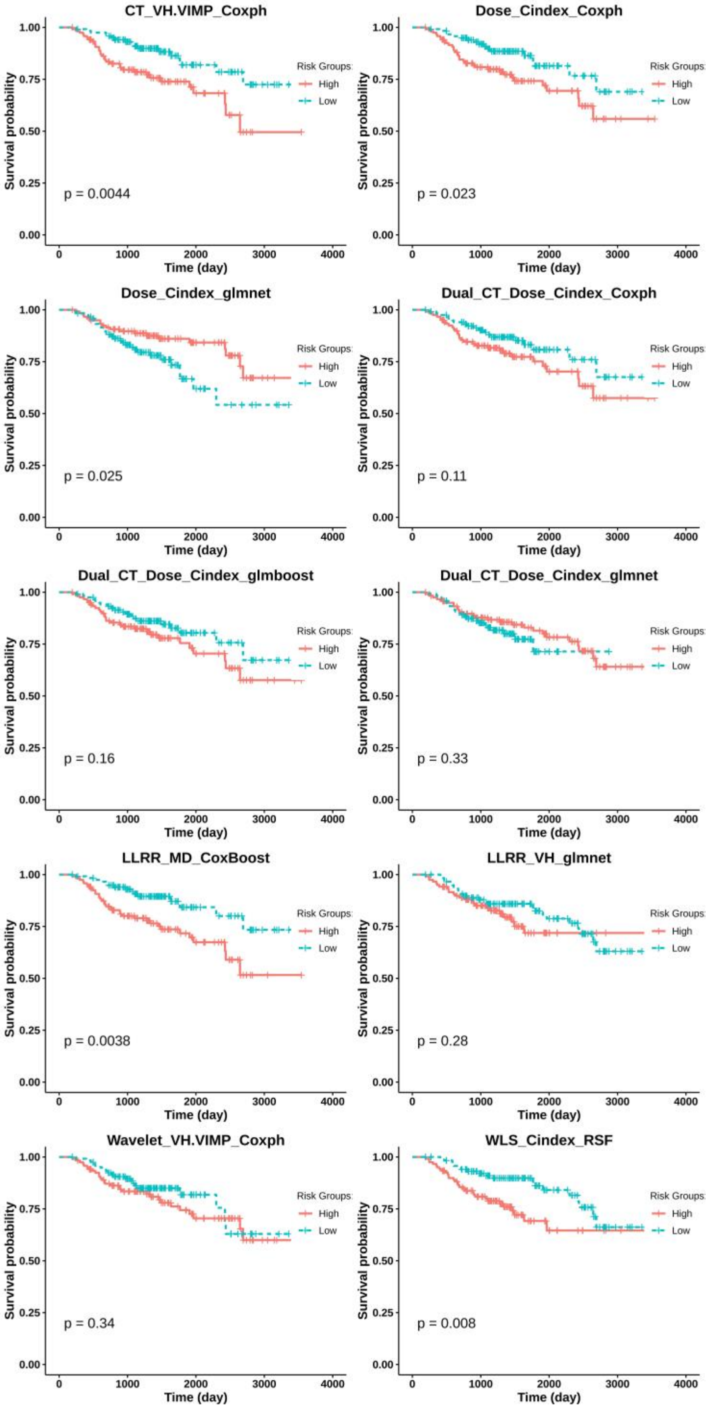

Supplementary figure 3. Kaplan-Meier curves for all investigated strategies.
